# scGraphVerse: a modular workflow for single-cell gene network inference

**DOI:** 10.1093/bioadv/vbag208

**Published:** 2026-07-25

**Authors:** Francesco Cecere, Daniela De Canditiis, Annamaria Carissimo, Claudia Angelini

**Affiliations:** Institute of Genetics and Biophysics, “Adriano Buzzati-Traverso”, Consiglio Nazionale delle Ricerche (CNR), Napoli, 80131, Italy; UOS Laboratori di Ricerca e Biobanca, UOC Ricerca Clinica e Traslazionale, AORN Santobono-Pausilipon IRCCS, Naples, 80129, Italy; Istituto per le Applicazioni del Calcolo “Mauro Picone”, Consiglio Nazionale delle Ricerche (CNR), Rome, 00185, Italy; Istituto per le Applicazioni del Calcolo “Mauro Picone”, Consiglio Nazionale delle Ricerche (CNR), Napoli, 80131, Italy; Istituto per le Applicazioni del Calcolo “Mauro Picone”, Consiglio Nazionale delle Ricerche (CNR), Napoli, 80131, Italy

## Abstract

**Motivation:**

Inferring gene networks from single-cell RNA sequencing data is challenging due to high sparsity, dimensionality, and technical noise. Current pipelines lack the multi-dataset integration and comprehensive post-processing analysis.

**Results:**

scGraphVerse is an R package that integrates multiple algorithms (GENIE3, GRNBoost2, ZILGM, PCzinb, and JRF) with extensive evaluation and visualization tools. Its modular workflow supports early, late, and joint integration strategies for multi-dataset analysis, providing standardized input/output interfaces and biological interpretation tools, including community detection, pathway enrichment, and literature mining. Benchmarking on simulated data showed model-based methods (PCzinb and ZILGM) perform well with limited sample sizes, while JRF performs best as the network size and dataset numbers increase. A PBMC case study demonstrates JRF’s ability to identify literature-supported regulatory communities across donors.

**Availability and implementation:**

The package is available in Bioconductor 3.22 at https://bioconductor.org/packages/release/bioc/html/scGraphVerse.html. Code and examples: https://github.com/ngsFC/scGV_analysis.

## Introduction

Understanding gene networks is crucial for interpreting how cells work. Single-cell RNA sequencing (scRNA-seq) technologies have revolutionized our ability to study gene networks at the cell-type level. However, single-cell expression data present unique computational and statistical challenges due to high sparsity, data dimensionality, technical noise, batch effects, and the need to distinguish between biological and technical signals (Trapnell *et al*. 2014, Chen and Mar 2018). Current network inference tools for single-cell data encompass multiple algorithms that take different data modalities as input (Badia-i Mompel *et al*. 2023), employing correlation analysis, regression models, probabilistic graphical models, dynamical systems, and deep learning to reconstruct the most precise gene network (Kim *et al*. 2023). Classical network inference tools for single-cell data include tree-based ensemble methods (GENIE3), gradient boosting algorithms (GRNBoost2), and graphical models specifically adapted for count data zero-inflated distributions (ZILGM and PCzinb) (Huynh-Thu *et al*. 2010, Moerman *et al*. 2019, Park *et al*. 2021, Nguyen *et al*. 2023). Although these methods work well individually, critical gaps remain in the field: (i) no single method consistently outperforms others (Pratapa *et al*. 2020, Hegde *et al*. 2025). (ii) Existing tools lack comparison capabilities and evaluation of the results. (iii) Each method has its own input/output format, which makes method comparison and selection difficult for beginners. (iv) Most approaches do not provide methods to integrate multiple single-cell expression datasets. (v) Post-hoc analysis from the inferred network to biological interpretation is usually provided thanks to external packages that require additional work and data manipulation. Current tools typically focus on individual-dataset network inference without systematic support for integrating information across multiple experiments. Multi-dataset scenarios are particularly challenging because of the heterogeneity and complexity of the data, as regulatory mechanisms may be conserved across cells under the same conditions.

We developed scGraphVerse to address these limitations by providing multiple algorithms and integrating various strategies, accompanied by extensive evaluation metrics. Through the scGraphVerse workflow, we provide an extensive interpretation of inferred biological networks, including community detection, pathway enrichment analysis, and literature mining.

## Materials and methods

### Overview and workflow

**Figure 1:**
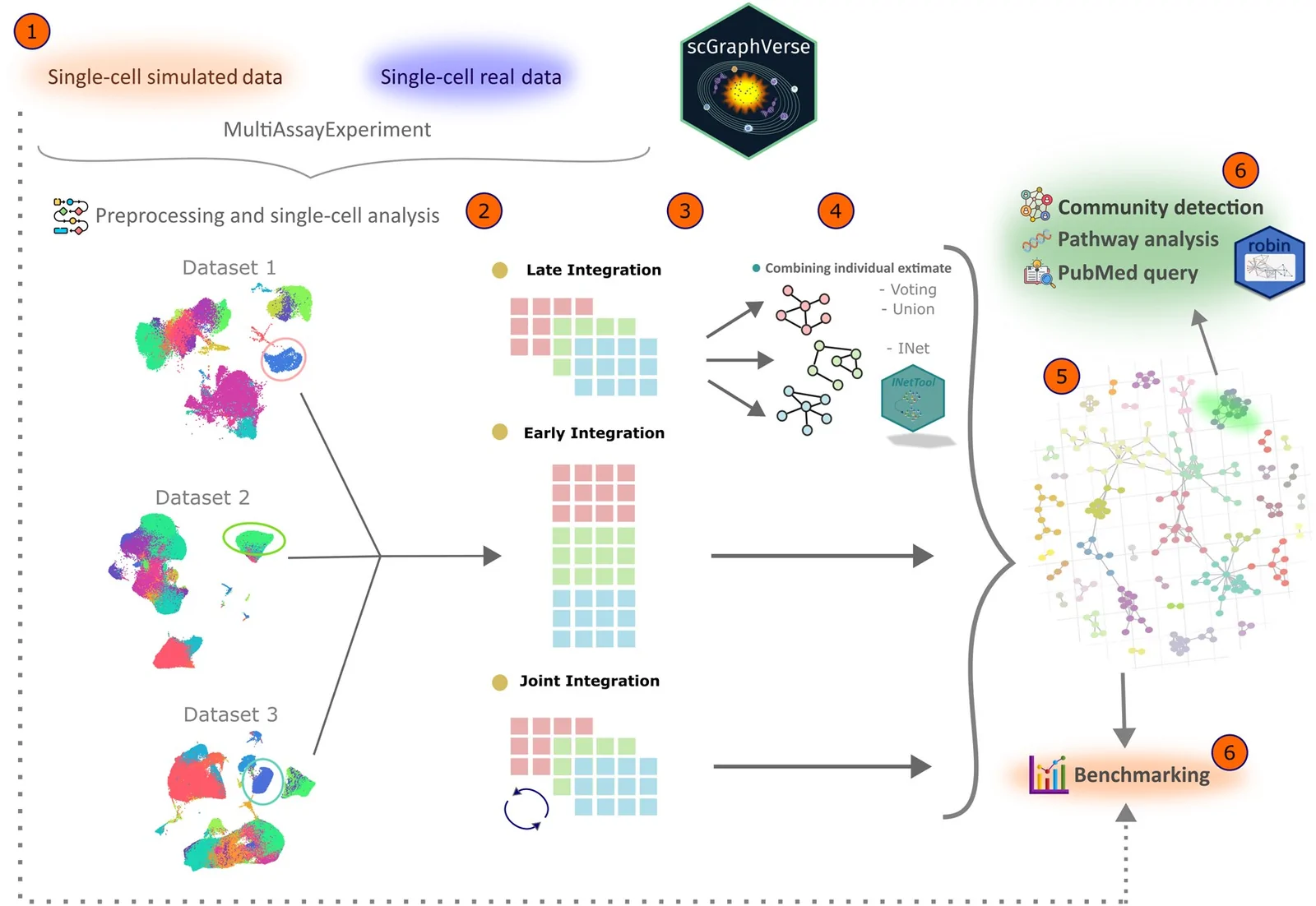
scGraphVerse modular workflow. The framework integrates five inference algorithms (GENIE3, GRNBoost2, ZILGM, PCzinb, and JRF) and supports three integration strategies (early, late, and joint) for handling multiple datasets. The pipeline provides a standardized input/output interface. It proceeds through network inference, edge selection, consensus building, and biological interpretation, using community detection and pathway enrichment analysis to offer a wide range of data inference and visualization options.

scGraphVerse workflow is shown in Fig. 1.

The workflow consists of six main modules: (i) *Input Data* as a MultiAssayExperiments containing raw scRNA-seq data. It can be created from multiple formats, including SingleCellExperiment, Seurat objects, count matrices, or a mixed list. (ii) *Flexible integration strategies* supporting early integration (dataset concatenation), late integration (independent dataset inference, followed by late consensus), and joint integration (simultaneous multi-dataset modeling). (iii) *Network inference* using five algorithms, each based on different assumptions. (iv) *Adaptive edges selection* procedure through null model-based thresholding for methods that do not have internal edge selection procedures. (v) *Consensus network construction* using voting, union, and graph-theoretic optimization (INet-Tool) to combine method predictions for the late integration strategy. (vi) *Comprehensive biological interpretation* tools, including community detection, pathway enrichment analysis, and validation through literature mining and STRINGdb.

Supplementary Section 1 provides a detailed description of the available modules.

### Notations and challenges

Let *K* denote the number of datasets and *p* be the number of genes of interest. For dataset *k*, we define the count matrix $X^{\left(k\right)}\in Z_{\geq 0}^{p\times n_{k}}$, which contains the expression values of *p* genes across $n_{k}$ cells, where $X_{ij}^{\left(k\right)}$ represents the read count of gene *i* in cell *j* within the dataset *k*. The goal is to infer a single undirected network G=(V,E) where vertices V={1,2,…,p} represent genes and edges E⊆V×V represent the shared relationships among the genes across the *K* datasets.

Single-cell count data exhibits several characteristics that challenges the network inference: (i) high data dimensionality because both the number of genes and the number of sequenced cells can be large, (ii) high sparsity in the raw data resulting from technical dropout events in which true gene expression is not detected, as well as from biological factors such as a lower mRNA capture efficiency or the absence of expression in that particular condition/cell, (iii) high sparsity in the network as only a limited set of edges is relevant, (iv) high level of noise due to overdispersion (i.e. variance of the counts exceeds the mean), technical noise associated with batch effects and amplification biases. Taken together, these factors make gene network inference from scRNA-seq data particularly challenging.

### Data processing of single-cell datasets

We assume that we have independently analysed *K* scRNA-seq datasets that correspond to a distinct experiment conducted under the same condition, time point, or involving the same cell-type population using standard pipelines [i.e. Seurat or Orchestrating Single-Cell Analysis with Bioconductor (Amezquita *et al*. 2020, Hao *et al*. 2023)], and the expression data are available (also in heterogeneous formats). We further assume that we have identified a specific cell type of interest (e.g. T or B cells) in each dataset and selected a set of genes associated with relevant pathways. For each dataset k∈{1,2,…,K}, scGraphVerse automatically extracts from the MultiAssayExperiment object the raw count matrix $X^{\left(k\right)}\in Z_{\geq 0}^{p\times n_{k}}$. The number of genes *p* is kept constant across all datasets, ensuring that scGraphVerse analyses a consistent set of genes of interest. Instead, the number of cells $n_{k}$ may vary across datasets due to different experimental designs. Each gene i∈{1,2,…,p} has an expression profile across all cells in dataset *k*, represented by the vector $x_{i}^{\left(k\right)}={\left[X_{i1}^{\left(k\right)},X_{i2}^{\left(k\right)},\ldots ,X_{in_{k}}^{\left(k\right)}\right]}^{T}$. This vector summarizes the heterogeneous expression pattern of gene *i* across the cellular population, capturing both the biological variability and technical noise of single-cell measurements.

scGraphVerse aims to reconstruct undirected gene networks G=(V,E) from the count objects $\left\{X^{\left(1\right)},X^{\left(2\right)},\ldots ,X^{\left(K\right)}\right\}$. When K=1, scGraphVerse performs inference in a single dataset mode. When K>1, scGraphVerse offers several integration strategies to perform inference with multiple datasets.

### Single dataset analysis

scGraphVerse supports inference and data exploration from a single scRNA-seq experiment. Since no integration step is required in this case, scGraphVerse performs network inference using one of the following algorithms: GENIE3, GRNBoost2, ZILGM, or PCzinb. The rationale and methodological details behind these methods are described in the Network Inference Methods section. The complete workflow follows all the steps described in Fig. 1, except for Module 2 (Integration strategy) and 5 (Consensus Network Construction).

### Integration strategies for multi-dataset analysis

scGraphVerse supports three integration strategies starting from a Bioconductor MultiAssayExperiment class objects:

- *Early Integration* concatenates the data before network inference, creating a single-dataset $X^{\left(\text{early}\right)}=[X^{\left(1\right)}|X^{\left(2\right)}|\cdots |X^{\left(K\right)}]$, under the assumption that biological mechanisms are shared across the same cell type. However, this strategy may be less appropriate for highly heterogeneous datasets or in presence of a significant batch effect. Then, inference is performed on $X^{\left(\text{early}\right)}$, as in the single-dataset mode. By effectively increasing the total number of cells *n*, this strategy enhances statistical power during network reconstruction.
- *Late Integration* performs network inference independently on each dataset within the MultiAssayExperiment, producing dataset-specific adjacency matrices $A^{\left(1\right)},A^{\left(2\right)},\ldots ,A^{\left(K\right)}$, which are then combined through consensus methods. This strategy preserves dataset-specific relationships while making a robust single network through ensemble aggregation.
- *Joint Integration* models multiple datasets within a unified inferential framework, simultaneously capturing the shared mechanisms and dataset-specific characteristics.

Through this framework, scGraphVerse enables the systematic exploration of inference methods and integration strategies, allowing researchers to select the most appropriate approach based on their experimental design, computational constraints, and biological hypotheses.

### Network inference methods

scGraphVerse integrates several inference algorithms. Each method is designed to address specific characteristics of single-cell data using different statistical approaches.

- *GENIE3* (Huynh-Thu *et al*. 2010) infers gene networks by identifying the neighbours of each gene independently. For each target gene *i*, it builds a predictive random-forest model $f_{i}:R^{p-1}\to R$ using the remaining *p* - 1 genes as predictors. Edges are identified by examining variable importance scores.
- *GRNBoost2* (Moerman *et al*. 2019) enhances the tree-based method described in GENIE3 by incorporating gradient boosting, thereby improving scalability through the Arboreto framework.
- *ZILGM* (Park *et al*. 2021) is based on a zero-inflated local graphical model. It estimates the neighbors of each gene using an $l_{1}$-penalized zero-inflated negative binomial regression, with the target gene as the response variable and the remaining p−1 genes as predictors. The regression coefficient $\Theta_{ij}$ represents the direct conditional dependence between genes *i* and *j*; a non-zero coefficient indicates the presence of an edge in the inferred network.
- *PCzinb* (Nguyen *et al*. 2023) is based on a zero-inflated global graphical model. The model assumes that genes follow a multivariate zero-inflated negative binomial (ZINB) distribution, with the network structure encoding conditional dependencies between genes. The algorithm begins with a complete graph on *V*. It tests marginal independencies between all pairs of nodes and removes edges when they are found. Then, for every pair of nodes, independence is tested conditionally on all subsets of cardinality one of the adjacency sets of the two nodes. This testing procedure iterates, increasing the size of the conditioning sets in turn, until it reaches its maximum limit or a user-imposed limit.
- *Joint Random Forests (JRF)* (Petralia *et al*. 2016) adopts a joint framework to analyse multiple datasets simultaneously. For each target gene *i*, it builds *K* predictive random forest models, one for each condition, where the target gene serves as the response variable and the remaining p−1 genes as predictors. To ensure that the models work jointly across multiple datasets, the algorithm enforces the use of the same splitting variables across the dataset-specific tree ensembles.

Table 1 provides an overview of the network inference algorithms. GENIE3, GRNBoost2, and ZILGM belong to the class of node-wise approaches, where network inference is performed by independently fitting a regression model for each gene (node), conditional on the expression of all other genes. PCZinb belongs to global-wise methods, as it performs inference simultaneously over the entire expression matrix. GENIE3, GNRBoost2, ZILGM, and PCZinb were originally designed for single-dataset analysis. Conversely, JRF belongs to the class of joint-inference methods and requires K>1 datasets. From a modeling perspective, GENIE3, GRNBoost2, and JRF are assumption-free methods, as they do not rely on any predefined statistical distribution of the expression data. In contrast, ZILGM and PCzinb are model-based approaches that assume gene expression counts follow a Zero-Inflated Negative Binomial (ZINB) distribution, explicitly modeling the overdispersion and sparsity inherent to single-cell data.

**Table 1:** Network inference methods and integration strategies in scGraphVerse.

Category	Method	Model-based	Algorithm	Single	Early	Late	Joint
Node-wise	GENIE3	×	Random Forest	✓	✓	✓	×
	GRNBoost2	×	Gradient Boosting	✓	✓	✓	×
	ZILGM	✓	Local Graphical Model (MLE)	✓	✓	✓	×
Global-wise	PCzinb	✓	Global Graphical Model (hypothesis test)	✓	✓	✓	×
Joint Modeling	JRF	×	Joint Random Forest	×*	×	×	✓

All methods accept a MultiAssayExperiment object as input. ✓ indicates that the corresponding integration strategy or modeling option is supported by the method, whereas a × denotes that it is not applicable. *JRF results using a single dataset are equivalent to those obtained with GENIE3.

### Matrix symmetrization for undirected graph

Regression-based gene network inference methods often produce asymmetric (weighted) adjacency matrices, in which the edge weight from gene *i* to gene *j*, $w_{ij}$, differs from that in the opposite direction $w_{ji}$. To obtain an undirected graph, we implemented a symmetrization function that transforms these directed networks into undirected ones. For each asymmetric (weighted) adjacency matrix $A\in R^{p\times p}$ representing *p* genes, the symmetrization process operates on pairs (i,j) and (j,i) where i≠j. The symmetric value $s_{ij}$ is computed according to the following rule:


(1) $$s_{ij}=s_{ji}=\{\begin{array}{cc} \text{max}(w_{ij},w_{ji}) & \text{if}\;w_{ij}=0\;\text{or}\;w_{ji}=0\\ f(w_{ij},w_{ji}) & \text{if}\;w_{ij}\neq 0\;\text{and}\;w_{ji}\neq 0 \end{array}$$

where f(·) is a function selected by the user (e.g. arithmetic mean, maximum, or minimum).

### Adaptive edge selection and thresholding

While the ZILGM and PCzinb algorithms directly provide binary adjacency matrices with non-zero entries indicating significant edges, GENIE3, GRNBoost2, and JRF provide variable importance scores for each edge pair. These scores depend on the specific method and datasets. Thus, inferring networks requires setting a threshold for converting the weighted adjacency matrix with importance scores into a binary form. scGraphVerse implements an adaptive null-model-based thresholding system that suggests an optimal cut-off. For each count matrix in the MultiAssayExperiment, *m* shuffled versions are generated by randomly permuting each gene’s expression across all $n_{k}$ cells, where $n_{k}$ denotes the total number of cells in the *k*-th dataset. This, preserves the distributional properties of the *p* genes while destroying the underlying regulatory relationships. Network inference is then performed on *m* shuffled matrices using the same method applied originally, generating null distributions of edge weights. The cutoff threshold is determined as $\tau_{k}=Q_{\alpha }(\{w_{ij}^{\left(s\right)}\})$, the α-quantile of edge weights from the shuffled networks. This approach ensures that the false positive rate is empirically controlled at level α under the null hypothesis of no regulatory relationships.

### Consensus approaches for late integration

When performing late integration strategies, scGraphVerse generates dataset-specific binary adjacency matrices that can be aggregated into a single, comprehensive consensus network. scGraphVerse constructs a consensus network through different approaches:

- *Voting approach:* For each potential edge (i,j), the support count $s_{ij}=\sum_{k=1}^{K}A_{ij}^{\left(k\right)}$ represents the number of datasets or methods predicting the edge. scGraphVerse constructs a voting consensus adjacency matrix as follows:
  $$A_{ij}^{\left(\text{cons}\right)}=\{\begin{array}{cc} 1 & \text{if}\;s_{ij}\geq \left\lceil \theta \cdot K\right\rceil \\ 0 & \text{otherwise} \end{array}$$

where θ∈(0,1] represents the voting threshold parameter.

- *Union approach:* This method includes any edge present in at least one of the input adjacency matrices:
  $$A_{ij}^{\left(\text{cons}\right)}=\underset{k=1,\ldots ,K}{\max}A_{ij}^{\left(k\right)}$$

This approach is equivalent to the voting approach with θ=1/K and maximizes network coverage; however, it also increases the false-positive rate.

- *INet-Tool:* scGraphVerse implements consensus methods through INet-Tool (which supports both weighted and binary adjacency matrices) (Policastro *et al*. 2025a), balancing edge inclusion with topological coherence.

### Community detection and biological interpretation

scGraphVerse assists the user not only with network inference but also with post-hoc analysis for community detection, pathway analysis, and literature mining validation.

- *Community Detection*: The inferred final network can be used as input for community detection algorithms to identify gene clusters of co-expressed or functionally related genes. To this end, scGraphVerse integrates all community detection algorithms available in igraph (Csardi and Nepusz 2006), including approaches based on modularity, divisive methods, statistical inference, dynamic processes, and alternative techniques. Moreover, scGraphVerse directly enables the selection of the algorithm that best fits, in terms of robustness of the identified community structure, the consensus network using the robin package (Policastro *et al*. 2021, 2025b).
- *Pathway Enrichment Analysis:* For each community containing at least $\tau_{\text{min}}$ genes (5 by default), scGraphVerse performs pathway enrichment analysis using both KEGG and Reactome databases, mapping gene symbols to Entrez identifiers (Xu *et al*. 2024, Yu 2024).
- *Literature Mining and STRINGdb validation:* To further support the user in the interpretation of the inferred network, scGraphVerse implements edge exploration through PubMed literature mining and STRINGdb (Winter 2017, Szklarczyk *et al*. 2023). For each predicted edge, queries are constructed and submitted to PubMed to retrieve information regarding the involved genes. Additionally, to evaluate biological significance, scGraphVerse compares the predicted networks to high-confidence STRINGdb interaction networks.

### Network visualization and data exploration

scGraphVerse provides a comprehensive set of plots and tables to facilitate the interpretation of inferred gene regulatory networks. The topology visualization enables the generation of network diagrams that support faceted comparisons across multiple datasets. For performance evaluation, the framework can compute receiver operating characteristic (ROC) curves and the corresponding area under the curve (AUC) values, using a user-provided ground-truth adjacency matrix as a reference. When an in-house reference network is unavailable, scGraphVerse benchmarks consensus networks against reference standards from STRINGdb and computes comprehensive performance metrics, including precision, TPR, and F1-score. While the goal of scGraphVerse is to infer a consensus network, ROC and AUC evaluations allow checking the quality of predicted edges across all possible thresholds, providing a measure of inference performance, i.e. independent from the threshold applied.

## Implementation and benchmarking

### Software architecture and standardization

We implemented scGraphVerse in R as a modular architecture that guarantees computational efficiency, reproducibility, and user-friendliness. scGraphVerse is available as a Bioconductor package.

### Unified interfaces and algorithms implementation

With scGraphVerse, we have integrated multiple gene network inference algorithms into a unified framework, i.e. well-harmonized with Bioconductor requirements, making the internal process transparent to the user. In addition, we introduced several technical innovations. In particular, we incorporated GRNBoost2 into R via the reticulate package (Allaire *et al*. 2023), since the original implementation was in Python within the Arboreto framework. We adapted the JRF, PCzinb, and ZILGM algorithms as internal R functions, modifying their original code to comply with Bioconductor guidelines and scGraphVerse interface requirements. Moreover, we provide a unified input/output interface via the infer_networks() function, which handles the algorithmic heterogeneity across the different inference methods. Using the create_mae() function, users can build a MultiAssayExperiment object from various single-cell data formats, including SingleCellExperiment, Seurat, or raw count matrices, with genes as rows and cells as columns. This process preserves consistent gene annotations across datasets and provides a harmonized input for infer_networks(). All assumption-free and the joint methods return edge lists, which we transform into binary adjacency matrices in the functions generate_adjacency(), symmetrize(), and cutoff_adjacency(). We store these matrices as SummarizedExperiment objects to ensure compatibility with downstream Bioconductor workflows. In contrast, model-based methods provide the binary matrices directly as SummarizedExperiment. We describe the internal architecture of scGraphVerse in the Supplementary Materials (Supplementary Section 1 and Fig. S1), and the main functions in Table S1. scGraphVerse is designed to work with transcriptomic data. Naive strategies for incorporating additional multi-omics layers (e.g. scATAC-seq and CITE-seq) into the workflow are briefly described in Supplementary Section 1.7. However, a full multimodal network inference framework is not currently implemented but represents a promising direction for future development.

### Visualization and data exploration functions

In scGraphVerse, we implemented a set of network visualization functions that enable researchers to translate computational predictions into biological knowledge. Table S2 summarizes the key functions available for network visualization, performance evaluation, and biological exploration.

We designed the plotg() function to explore the network topology and community structure, while plotROC() and compare_consensus() offer quantitative performance assessments against a biological reference. In addition, with the community_path() function, we perform community detection and pathway enrichment analysis to link gene modules with biological functions. All those functions take a Bioconductor SummarizedExperiment object containing the inferred networks as input. Using these functions, we facilitate the interpretation of complex network inference results by leveraging several bioinformatics databases (STRINGdb, KEGG, Reactome).

### Parallelization and performance optimization

To speed up execution, we implemented parallelization strategies through BiocParallel integration (Morgan *et al*. 2025). While GENIE3 and ZILGM utilize their internal parallelization, in scGraphVerse, we parallelized GRNBoost2 and PCzinb across multiple datasets, assigning each dataset to a separate core. However, the current version of scGraphVerse does not include any additional parallelization for JRF.

### Simulation framework and benchmarking

We illustrate the performance of scGraphVerse through an extensive simulation study, in which networks with known structures were retrieved from STRINGdb as ground truth while varying the number of cells, genes, and noise level. Figure S2 illustrates examples of the resulting networks for different values of *p*, corresponding to varying numbers of genes. We provide a detailed description of the simulation data generation procedure in Supplementary Section 2.

We designed three simulation scenarios. In the first scenario, we compared the performance of all the methods by generating K=3 count matrices, fixing the number of cells at n=100 and varying the number of genes *p* from 100 to 500. In the second scenario, we evaluated scalability with respect to both *p* and *n*, fixing K=3 and varying *p* from 100 to 700 and *n* from 100 to 500. In the third scenario, we evaluated performance as we increased the number of datasets to K=5 count matrices, fixing n=100 and p=500. In practice, we do not fix the number of genes *p*; e.g. when we write p=100, we refer to a network with approximately 100 genes (p∼100). For computational reasons, in the second and third scenarios, we restricted the comparisons to assumption-free and joint methods, i.e. GENIE3, GRNBoost2, and JRF. In all settings, we used distinct condition-specific parameters to mimic experimental heterogeneity. Table S3 summarizes the parameters used for data simulation in all scenarios. In the simulation scenarios, we evaluate the algorithms by calculating several measures that summarize edge-, network-, and community-level performance (see Supplementary Section 3) (Sokolova and Lapalme 2009, Chicco and Jurman 2020, Newman 2010).

For each algorithm, we performed inference over 10 independent realizations, taking the mean values for each type of measure. We performed all simulations on a computing system equipped with an Intel Core i9-9900K processor (8 cores, 16 threads, 3.60 GHz base frequency, and up to 5.00 GHz boost), 64 GB of RAM, and an Ubuntu 20.04.1 LTS operating system (based on the Linux kernel 5.15.0–139-generic). Moreover, we conducted all analyses in R version 4.5.1, with the BiocParallel backend configured for parallel processing across 15 CPU cores. The code to reproduce the simulation analysis is available in the GitHub repository (see the “Data availability” section).

## Results

### Results for simulation data

#### Performance on edge inference

To evaluate the overall performance of the gene network algorithms in the first simulation scenario, we benchmarked all methods under both early and late integration strategies. For the late integration analysis using GENIE3, GRNBoost2, ZILGM, and PCzinb, we report results obtained with the consensus “voting” approach (with θ=0.50), as it consistently achieved better performance in terms of precision, MCC, and F1-score for both n=100 and n=500. We provide comparisons with alternative consensus strategies in Supplementary Section 4 and Fig. S3.

**Figure 2:**
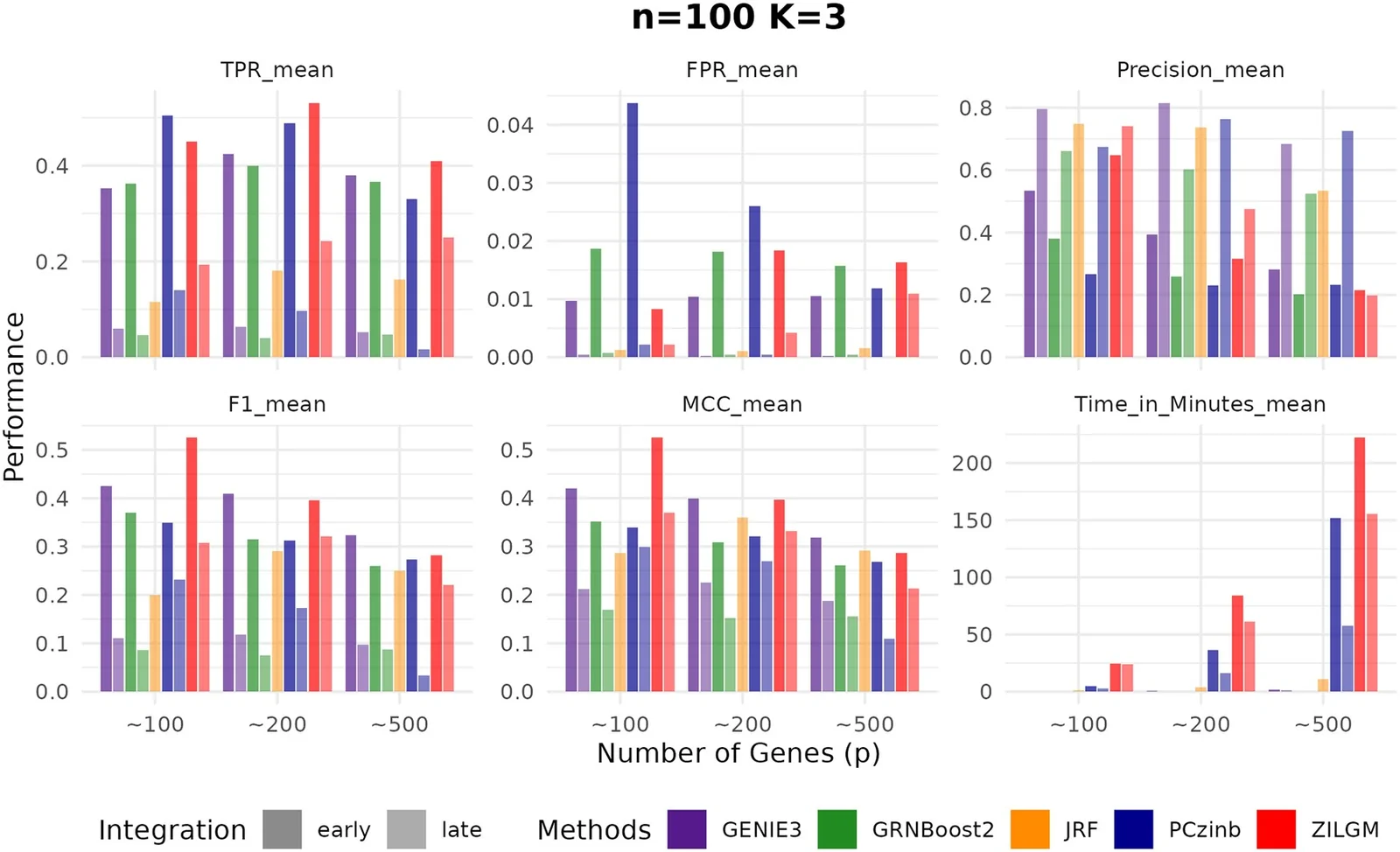
First simulation scenario: Edge-level measures for *n *= 100 cells. Comparison of all the methods gene network inference performance indexes increasing *p* genes (p=100−500) when *n *= 100 cells and *K *= 3 datasets. Colours represent inference algorithms: GENIE3 (purple), GRNBoost2 (green), JRF (orange), ZILGM (blue), and PCzinb (red). The color shades denote the Integration strategies, with early integration (darker) and late integration (lighter).

Considering n=100 and varying *p* from ∼100 to ∼500, we noticed differences among the assumption-free and model-based methods (Fig. 2). For model-based methods, PCzinb exhibits a low TPR under late integration, which further decreases with increasing *p*, while the FPR remains very low and precision remains high; F1 and MCC also decrease accordingly. Under early integration, TPR is higher for smaller networks (p∼100), but both TPR and FPR decrease as *p* increases, with precision remaining constant and F1 and MCC remaining moderate. ZILGM achieves a higher TPR than PCzinb under late integration, but at the cost of a higher FPR and lower precision. Early integration further increased both TPR and FPR, whereas precision decreased slightly in larger networks. However, both PCzinb and ZILGM exhibit severe computational scalability issues, with computing times rising from about 30 minutes for small networks (∼100 genes) to over 200 minutes for large ones (∼500 genes). Assumption-free methods GENIE3 and GRNBoost2 achieve a low TPR with a very low FPR under late integration, where they also exhibit higher precision but lower F1 and MCC scores. Under early integration, TPR increases substantially, accompanied by a moderate rise in FPR, whereas precision decreases slightly. However, both F1 and MCC improved, indicating better overall edge recovery despite a higher number of false positives. Finally, the joint method (JRF) consistently performs well, maintaining low FPR and high precision, and outperforms late integration assumption-free methods. As model-based algorithms are advantageous for analysing relatively small expression matrices, where capturing global dependencies becomes important (p∼100), we observed that they become computationally demanding when the analysis requires the comparison and integration of multiple, larger datasets. Considering this, we limited the benchmarking of the remaining scenarios to assumption-free and joint methods.

**Figure 3:**
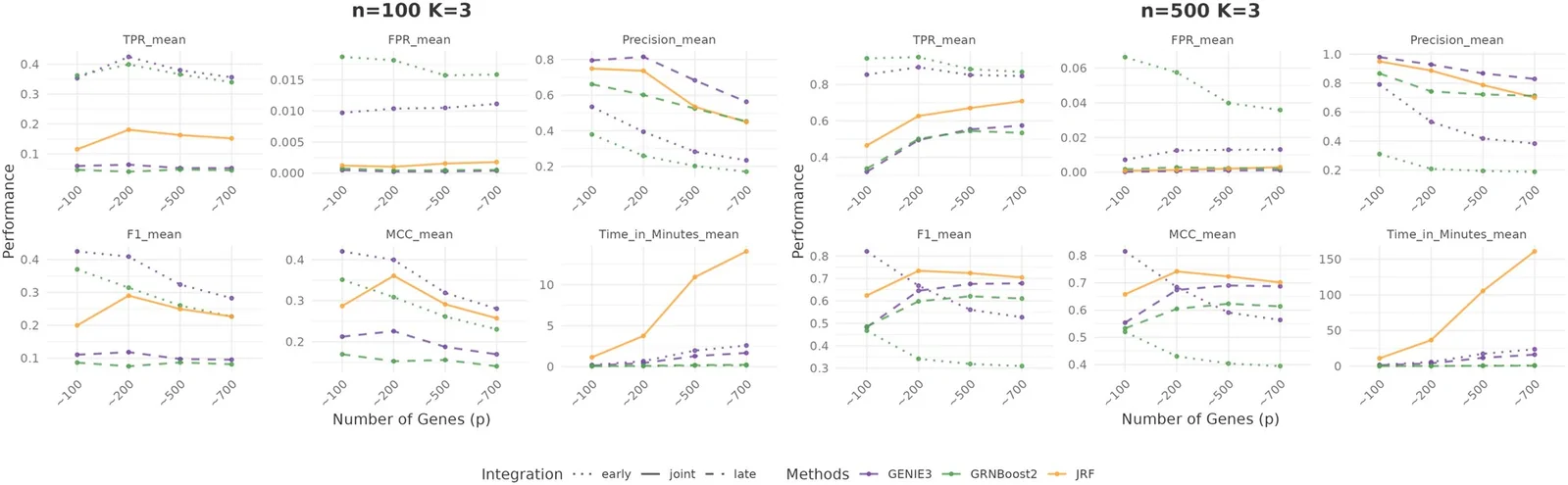
Second simulation scenario: Edge-level measures for n=100−500 cells. Comparison of gene network inference performance indexes increasing *p* genes (p=100−700) when *n *= 100 (left) and *n *= 500 (right) cells and *K *= 3 datasets. Colours represent inference algorithms: GENIE3 (purple), GRNBoost2 (green), and JRF (orange). The line types denote the integration strategies as early integration (dotted), late integration (dashed), and joint integration (solid).

In the second scenario, we performed the benchmarking analysis by increasing the network sizes (100–700 genes) while keeping the number of cells and datasets fixed. Figure 3 summarizes the average edge-level measurements for n=100 and n=500. We found that JRF consistently outperformed the other algorithms across all evaluation indices. For n=100 cells and increasing *p*, we observed that JRF achieves a TPR ranging from 0.12 (p=100) to 0.18 (p=200), remaining constant at 0.15 for p=700. In contrast, the late integration approaches based on GENIE3/GRNBoost2 never exceeded a TPR of 0.06. When compared with early integration strategies, JRF exhibited lower TPR values (∼0.15 versus ∼0.35), but maintained substantially lower FPR (0.002 versus 0.01–0.02). Consistently, we observed that JRF showed higher precision, starting at 0.75 (p=100) and remaining at 0.45 (p=700), whereas the early and late integration strategies achieved $<$0.30 and $<$0.60, respectively. Regarding the F1 score, JRF reached a peak of 0.28 at p=200, maintaining values above 0.22 up to p=700, making its performance comparable to early integration and superior to late integration approaches. Moreover, JRF achieved a higher MCC (0.35) than any late integration method. For n=500, we observed a further improvement with TPR ranging from 0.47 to 0.67, precision from 0.85 to 0.95, F1 scores above 0.62–0.73, and MCC remaining stable at ∼0.70, all achieved with consistently low FPR.

#### Performance on network topology and community structure

**Figure 4:**
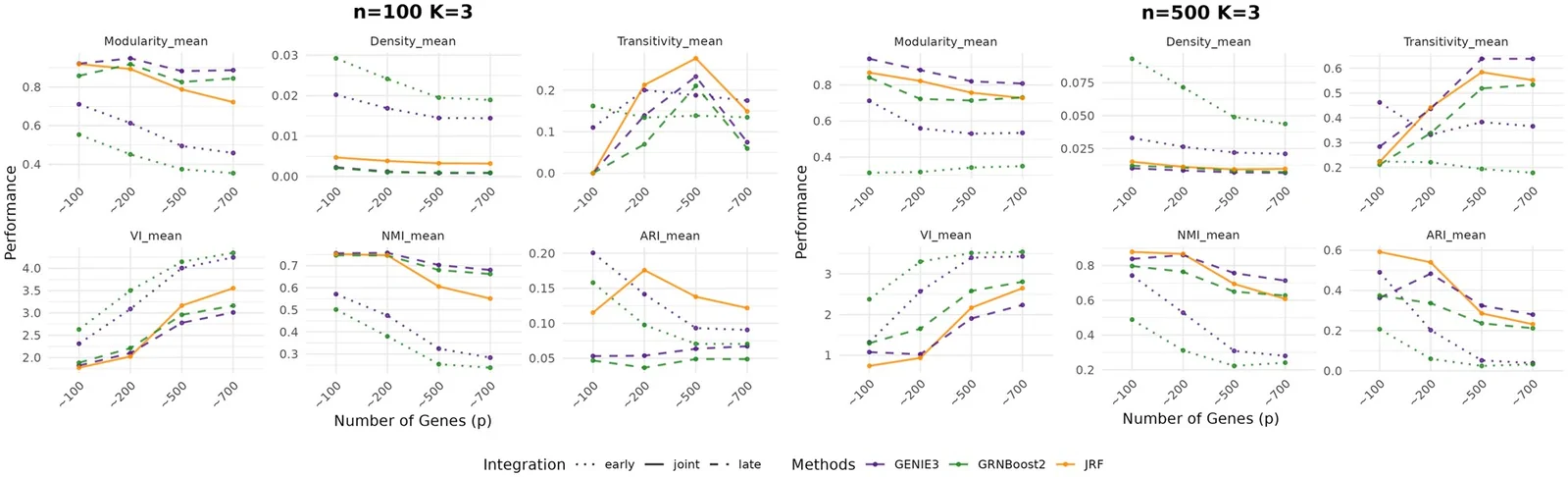
Second simulation scenario: Network and Community measures for n=100−500 cells. Comparison of gene network inference performance indexes increasing *p* genes (*p *= 100–700) with *n *= 100 (left) and *n *= 500 (right) cells and *K *= 3 datasets. Colours represent inference algorithms: GENIE3 (purple), GRNBoost2 (green), and JRF (orange). The line types denote the integration strategies: early integration (dotted), late integration (dashed), and joint integration (solid).

We observed that JRF preserved both the network topology and community structure across all tested conditions (Fig. 4). Specifically, JRF maintained high modularity values (>0.85 at p=100, ∼0.75 at p=700), while GENIE3/GRNBoost2 algorithms drop from 0.60 to 0.40. Moreover, JRF produces very sparse networks (density range: 0.004–0.002), thereby preventing spurious edges, unlike those observed in early integrative frameworks (density range: 0.02–0.03). Furthermore, we found that our community structure measures support JRF’s performance, with NMI values of approximately 0.75, decreasing to 0.55 as the network size increases. Similarly, ARI reaches a maximum of 0.18 for JRF and remains above 0.12, whereas the other approaches stay below 0.10. Finally, we identified consistently lower VI scores for JRF, indicating better agreement with the ground-truth community structure. When considering edge-level measures at n=500, we observed that the late and joint integration methods outperformed the early integration strategy.

#### Computational performance and scalability

JRF’s computational cost represents a reasonable trade-off for its significant performance gains (bottom-right for n=100 and n=500 in Fig. 3). We observed that JRF required between 1.2 and 15 minutes across networks of 100–700 genes, which was competitive with GENIE3 (0.5–2 minutes), while significantly outperforming both methods in terms of accuracy metrics. We noted that GRNBoost2 had the fastest runtime (0.05–0.2 minutes); however, it achieved this at the cost of network reconstruction quality across all evaluation metrics. However, GENIE3 and GRNBoost2 become practical alternatives when the dataset size is particularly challenging.

#### Performance at multi-dataset integration

**Figure 5:**
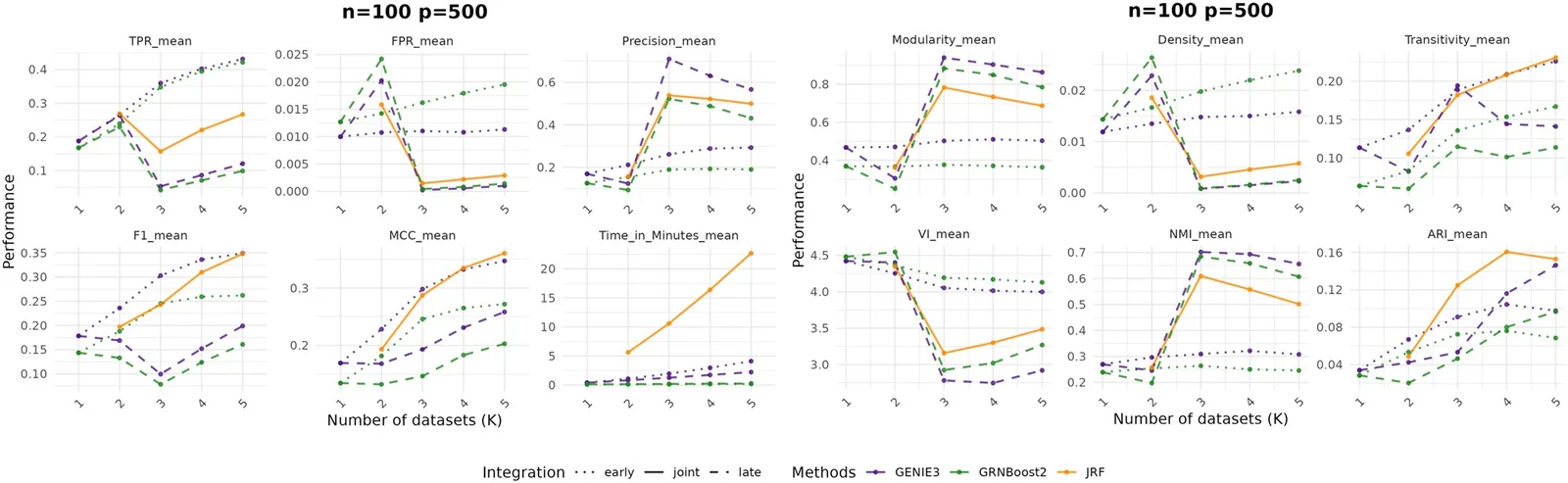
Third simulation scenario: Edge-level (left) and Network and Community (right) metrics for *n *= 100 cells and *p *= 500 genes. Comparison of gene network inference performance metrics increasing *K* from 1 to 5 datasets. Colors represent inference algorithms: GENIE3 (purple), GRNBoost2 (green), and JRF (orange). The line types denote the integration strategies: early integration (dotted), late integration (dashed), and joint integration (solid).

In the third scenario, we scaled the analysis from K=1 to K=5 while keeping the network size fixed (n=100, p=500), revealing JRF’s enhanced capability in both network reconstruction and community structure preservation (Fig. 5). In particular, we observed that TPR increased to 0.26 at K=5, with a substantial improvement in precision from 0.18 to 0.55, particularly for datasets with K>3. This translated into consistent gains in F1 score (from 0.20 to 0.35) and MCC (from 0.19 to 0.35), indicating that joint modeling becomes more powerful as *K* increases. Our network topology analysis also highlights JRF’s strengths in multi-dataset scenarios (Fig. 5). We found modularity scores between 0.75 and 0.80 and a low network density (∼0.005), indicating that JRF effectively avoided the edge overestimation typical of other methods. Transitivity also increased, reaching 0.22 at K=5, reflecting better local clustering as the number of datasets grows. Community structure metrics were consistent with these findings (Fig. 5): NMI remained high (∼0.6), while ARI improved significantly beyond K=3 (0.16 versus $<$0.10 for other methods). Moreover, VI decreased notably for JRF at higher *K* values, indicating more accurate community detection. In contrast, we observed that GENIE3 and GRNBoost2 showed little to no improvement across datasets under both early and late integration, plateauing at lower performance levels, regardless of the value of *K*. As shown in the bottom-right panel of Fig. 5, JRF’s computational cost scaled linearly, ranging from 5 to 23 minutes as *K* increased from 2 to 5.

### Results for real data

#### Case study 1: PBMC results

**Figure 6:**
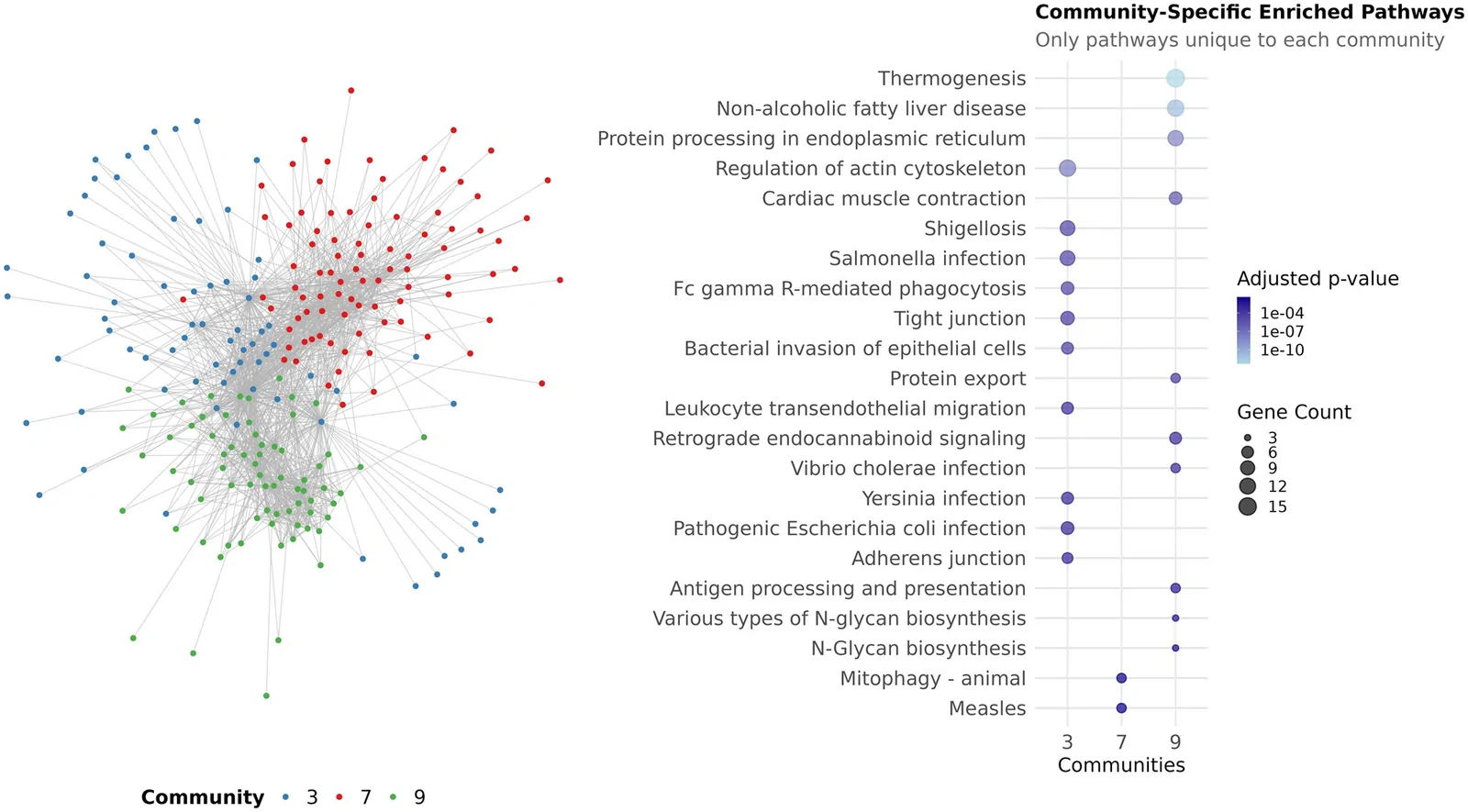
Network and pathways: Left panel: Network visualization of gene relationships identified by the JRF algorithm from B cell scRNA-seq data. Nodes represent genes and edges represent regulatory interactions. Node colours indicate community membership. Right panel: KEGG pathway enrichment analysis for communities. Dot colour intensity indicates the statistical significance of enrichment. Dot size is proportional to the number of genes from each community present in the pathway.

To illustrate scGraphVerse’s ability to analyse real data, we applied it to publicly available single-cell RNA-seq data from peripheral blood mononuclear cells (PBMCs) from the Single Cell Atlas (www.singlecellatlas.org). We downloaded the dataset using the download_Atlas() function from the specified URL, which contained scRNA-seq data from adult cardiovascular system blood samples. From the complete PBMC atlas, we extracted B cell populations from three donor samples (GSM5073064_CHD1_PBMC, GSM5073066_CHD3_PBMC, GSM5073068_CHD5_PBMC) using Seurat subsetting based on the CELL_TYPE metadata annotation column. To extract the primary biological signal, we selected the top 600 most highly expressed genes using the selgene() function with the following parameters: top_n = 600, cell_type = “B_cell”, remove_rib = TRUE, and remove_mt = TRUE to exclude ribosomal and mitochondrial genes that could affect biological interpretation. We applied the JRF algorithm to the MultiAssayExperiment, which contains three B-cell datasets, to infer a network used for community detection and KEGG pathway analysis. To ensure a robust interpretation, we focused our analysis on communities containing at least 70 genes. Performing network analysis on B cell scRNA-seq data, we identified three main regulatory communities (Communities 3,7,9) containing at least 70 genes each (Fig. 6, left panel). To investigate the community’s biological functions, we performed KEGG pathway analysis, which revealed distinct community-specific roles in B cell regulation (Fig. 6, right panel). Considering the pathways uniquely enriched, we highlight cytoskeletal regulation and cellular motility pathways (Community 3), protein processing and metabolic pathways (Community 9), and metabolic homeostasis and stress response through mitochondrial quality control (Community 7), which recapitulate the main functions of B-cells. To better study the role of specific genes in community-specific pathways, we performed literature mining validation, focusing on genes absent from the STRINGdb physical interaction database (Supplementary Section 5 and Table S4). Considering the predicted edges, we provide strong evidence from the literature, with highly cited edges that recapitulate relevant cellular functions.

#### Case study 2: Medulloblastoma dataset

**Figure 7:**
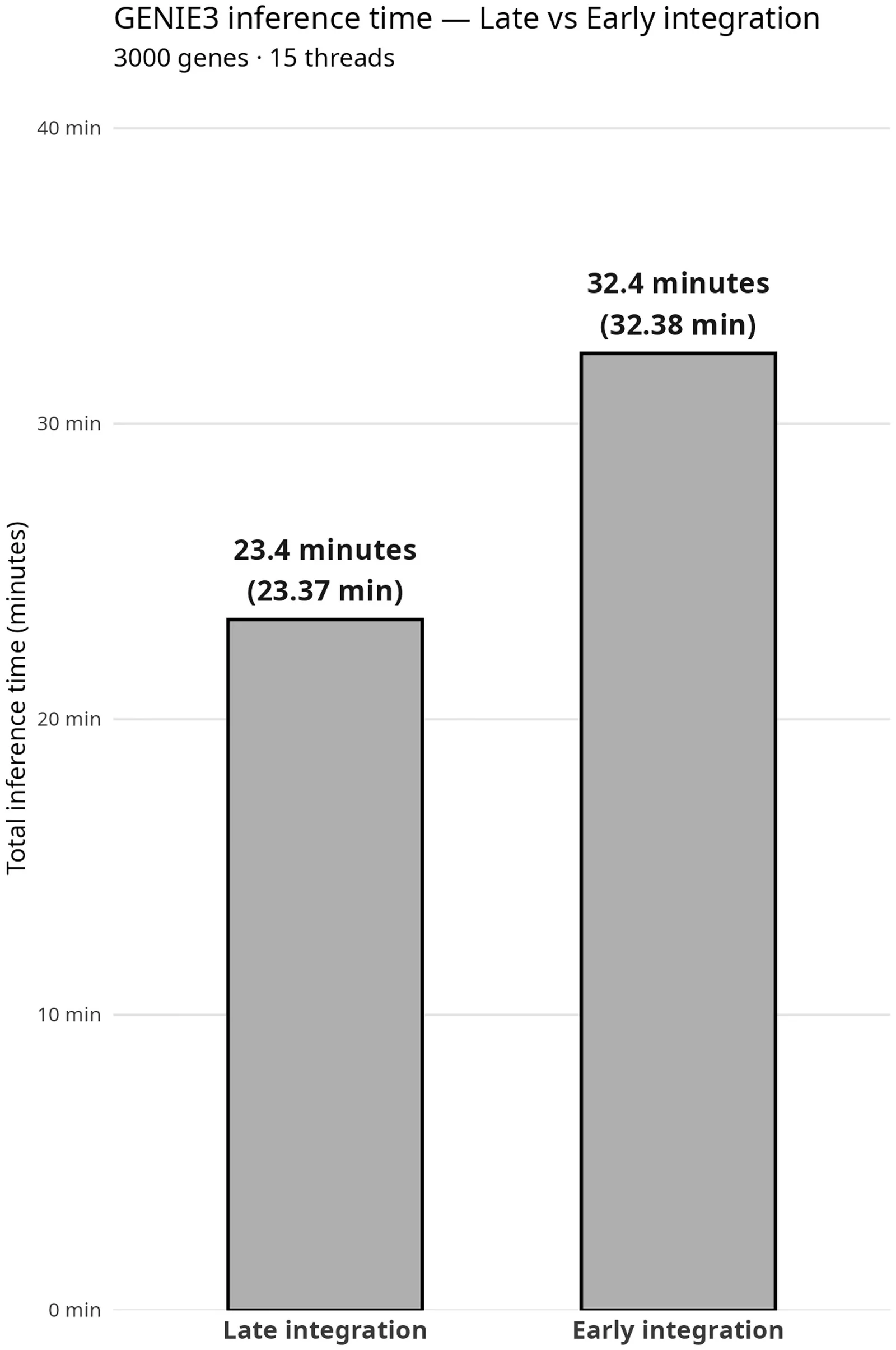
Network inference computational time under late and early integration strategies: GENIE3 inference time (minutes) for late and early integration strategies on medulloblastoma scRNA-seq data (3000 genes, 525 cells, 15 cores).

To benchmark the scalability and biological relevance of the scGraphVerse framework, we applied gene network inference to scRNA-sequencing data from two pediatric medulloblastoma patients (SCPCS000136, SCPCS000139) (Lee *et al*. 2024, Hawkins *et al*. 2024). We selected the top 3000 expressed genes, excluding mitochondrial, ribosomal, and MALAT1 transcripts. We ran the full scGraphVerse pipeline using GENIE3 (15 cores) as the inference method under two integration strategies: late integration (consensus voting) and early integration. Both the analyses were completed within reasonable computational time (23 and 34 minutes, respectively) (Fig. 7). For both the networks, we call the gene communities and perform pathway analysis revealing key mechanisms for medulloblastoma biology, including: IgSF CAM signaling, Focal adhesion, Ubiquitin mediated proteolysis, detected in both integration strategies and all related to proliferation, invasion, and metastasis (Supplementary Section 6 and Fig. S4).

## Discussion

We present scGraphVerse, a comprehensive R package for gene network analysis that addresses the critical gap in integrating multiple datasets for network inference. Traditional methods operate on single matrices, limiting their ability to capture shared regulatory mechanisms across multiple experiments. Our framework implements three distinct integration strategies—early, late, and joint—enabling researchers to systematically explore how different approaches affect network reconstruction quality and to use such approaches to analyse their data. While early integration requires stronger assumptions of homogeneity and absence of batch effects, the other two integration strategies are more robust to inter-dataset variability: late integration models each dataset independently before combining, while joint integration infers the network simultaneously across datasets. Altogether, these approaches provide a range of strategies suitable for different experimental settings. As a key point, we provide a standardized input/output interface that works with expression data stored in a MultiAssayExperiment. By providing this unified interface, we eliminated the need for complex data conversion and harmonized scGraphVerse with other Bioconductor packages. Moreover, in the current version, we included five inference algorithms (GENIE3, GRNBoost2, ZILGM, PCzinb, and JRF) within a unified computational architecture, enabling a systematic comparison of methods with identical preprocessing, evaluation metrics, and validation procedures. To address the challenge of converting continuous importance scores into binary networks, we implemented an adaptive null model-based thresholding system that automatically determines optimal cutoffs while controlling false-positive rates. While model-based methods are more effective in analysing smaller datasets, our evaluation revealed that the joint algorithm is the most efficient method for multi-dataset network inference, outperforming traditional approaches across several evaluation metrics, particularly as the number of datasets increases. As a significant advantage in downstream processes, we integrate several tools for community and pathway analysis within a single computational environment, enabling exploration and analysis of the existing literature on network edges. We demonstrated this particularly in the PBMC case study, where, with JRF, we successfully extracted biological information from complex scRNA-seq data across multiple donors, identifying four main regulatory communities with distinct functional characteristics, including adaptive immune responses, cellular stress and energy metabolism, cytoskeletal regulation, and the protein synthesis machinery. Even more, using medulloblastoma datasets, we highlight the scalability of our framework up to 3000 genes, identifying communities that capture pathways directly relevant to medulloblastoma cancer progression. By integrating these tools, we eliminate the need for multiple pipelines and minimize potential errors throughout the analysis. A current limitation of scGraphVerse is that all inference methods produce undirected symmetric networks, which cannot explain the directionality of regulatory relationships. Thus, future developments will explore Bayesian and causal inference approaches to infer directed regulatory relationships from multiple datasets, including transcription factor binding information and multimodal data, such as scATAC-seq. Finally, we designed the modular architecture of scGraphVerse to facilitate future extensions with other network inference algorithms while maintaining compatibility standards.

## Supplementary Material

vbag208_Supplementary_Data

## Data Availability

The scGraphVerse R package is available in the Bioconductor 3.22 release https://bioconductor.org/packages/release/bioc/html/scGraphVerse.html. All simulation data, analysis code, and examples are available at https://github.com/ngsFC/scGV_analysis.
