# Supplementary material for: scGraphVerse: a modular workflow for single-cell gene network inference
Source: Bioinform Adv. 2026 Jul 25;6(1):vbag208. doi: 10.1093/bioadv/vbag208 (PMC13524424; doi:10.1093/bioadv/vbag208)
Supplement: Supplementary file 1 [file vbag208_supplementary_data.pdf]

scGraphVerse: Supplementary Materials

Francesco Cecere, Daniela De Canditiis, Annamaria Carissimo, Claudia Angelini

July 6, 2026

Contents

1 scGraphVerse Workflow Overview **2**
- 1.1 Module 1: Input Data . . . 3
- 1.2 Module 2: Integration Strategy . . . 4
- 1.3 Module 3: Network Inference . . . 4
- 1.4 Module 4: Edge Selection . . . 5
- 1.5 Module 5: Consensus Network Building . . . 5
- 1.6 Module 6: Analysis & Validation . . . 6
  - 1.6.1 Integration & Inference Performance Evaluation . . . 6
  - 1.6.2 Network Visualization & Analysis . . . 7
- 1.7 Multimodal Integration and future extensions . . . 8

2 Data Simulation **8**
- 2.1 Ground Truth Network Construction . . . 8
- 2.2 Zero-Inflated Count Data Simulation . . . 9

3 Performance Evaluation Framework **10**
- 3.1 Edge-Level Performance Indices . . . 10
  - 3.1.1 Sensitivity and Specificity . . . 11
  - 3.1.2 F1-Score . . . 11
  - 3.1.3 Matthews Correlation Coefficient . . . 11
- 3.2 Network-Level Performance Measures . . . 11
- 3.3 Community Structure Performance Measures . . . 12

4 Consensus approaches for late integration **13**

5 Case study 1: Edge mining **13**

6 Case study 2: Medulloblastoma network inference **13**

1 scGraphVerse Workflow Overview

scGraphVerse provides a modular framework for gene network inference from single-cell gene expression data, offering various algorithms that support multiple integration strategies, as well as tools for post-inference analysis, visualization, and interpretation. It internally manages all data formatting and preprocessing required by the integration and inference methods, enabling the user to perform complex analyses through a few sequential function calls. Moreover, scGraphVerse is a Bioconductor package that standardizes the input/output data structures to ensure full compatibility and interoperability with the Bioconductor ecosystem and packages. In particular, it uses the MultiAssayExperiment as input to the network inference function and the SummarizedExperiment to store the inferred networks.

The scGraphVerse workflow consists of six interconnected modules that guide the user from raw count data to gene network inference, community detection, and pathway analysis. Figure S1 provides a detailed scheme of the internal architecture and data flow, while Table S1 provides the main functions.

Table S1: Overview of scGraphVerse main functions.

Category	Function	Description
**(1) Input Data**<br>*Automated handling of multiple formats*	`create_mae()`	Creates MultiAssayExperiment from lists of count matrices, Seurat objects, or SingleCellExperiment objects for integrated analysis
	`selgene()`	Selects top-expressed genes from count objects with optional cell-type and gene filtering (removing mitochondrial and/or ribosomal genes)
**(2) Flexible Integration Strategies**<br>*Early, late, joint*	`earlyj()`	Combines datasets inside the MultiAssayExperiment for early integration strategies
**(3) Network Inference**<br>*modular inference methods*	`infer_networks()`	Core function implementing five algorithms: GENIE3, GRNBoost2, ZILGM, PCzinb and JRF
	`generate_adjacency()`	Converts GENIE3, GRNBoost2 and JRF outputs to standardized weighted adjacency matrices
	`symmetrize()`	Symmetrizes adjacency matrices (mean, max, min) to handle asymmetric network outputs
	`init_py()`	Initializes Python environment and loads required modules (arboreto,pandas, numpy) for GRNBoost2
**(4) Edge Selection**<br>*via null models*	`cutoff_adjacency()`	Applies adaptive thresholds using null model approach: generates shuffled matrices, infers networks, calculates quantile-based cutoffs
**(5) Consensus Network Construction**<br>*for late integration*	`create_consensus()`	Builds consensus networks through: voting, union, or INet-Tool
**(6) Comprehensive Biological Interpretation**	`community_path()`	Detects gene communities using multiple algorithms, performs KEGG/Reactome pathway enrichment
	`edge_mining()`	Performs systematic literature validation by querying PubMed
	`stringdb_adjacency()`	Retrieves high-confidence protein-protein interactions from STRINGdb API to construct reference networks for validation

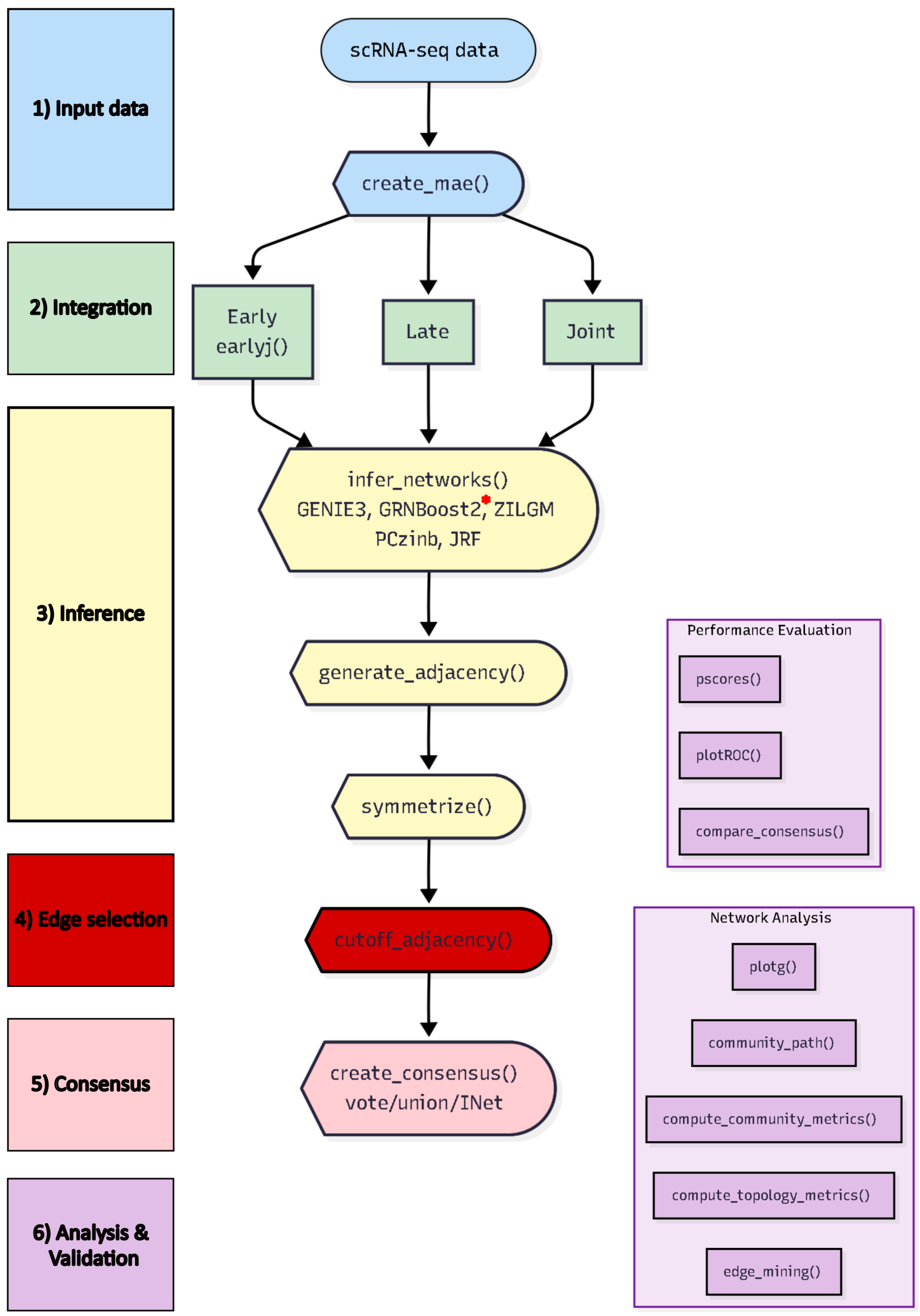

Figure S1: **scGraphVerse function architecture** The scGraphVerse workflow is implemented by combining the main functions across several steps. The left panel refers to the specific module where the function is used. The central panel illustrates the entire workflow, while the right panel displays the specific functions for exploring the output and performing further analysis and validation. * Note that to run the inference with GRNBoost2 as a method, the user should prepare the Python environment using the `init_py()` function.

1.1 Module 1: Input Data

The workflow begins by preparing one or more scRNA-seq datasets to provide well-structured input for further analyses. scGraphVerse accepts various data formats, including `SingleCellExperiment` objects, `Seurat` objects, or raw count matrices. To proceed with network inference, the user must first organize the data into a unified structure by creating a `MultiAssayExperiment` (MAE) ob-

ject using the `create_mae()` function (see Figure S1). Then, depending on the number of datasets in the MAE object, scGraphVerse proceeds with inference and analysis, offering different options.

1.2 Module 2: Integration Strategy

When the MAE object contains a single dataset, the user can choose the specific inference method directly, skipping the integration strategy step. By contrast, when multiple datasets are present, the user can choose an integration strategy. scGraphVerse supports three integration strategies:

- **Early Integration**: scGraphVerse merges expression data across samples, increasing the number of cells before running network inference.
- **Late Integration**: scGraphVerse infers networks independently for each dataset, then combines results at the adjacency matrix level through a consensus method.
- **Joint Integration**: scGraphVerse uses the Joint Random Forest method to learn from multiple datasets simultaneously.

The choice of integration strategy depends on how the MAE object is organized and the specific inference method used. Therefore, for early integration, the user needs first to merge expression data stored in the MAE object using the `earlyj()` function and then run the network inference. Note that the `earlyj()` function is the only currently available function in this module and is required only for guiding scGraphverse through early integration (see Figure S1). For late and joint integration, no additional preprocessing is required at this stage, and the user can directly use the MAE object created in Module 1 to proceed with network inference.

Given the choice of integration strategy, the data internal organization and the rest of the process are entirely transparent to the user, as the decision between late and joint approaches depends on the inferential method, as described in the next section.

1.3 Module 3: Network Inference

This module represents the core network inference and supports multiple inferential algorithms. According to Figure S1, it includes the main function `infer_networks()`, and two additional functions (`generate_adjacency()` and `symmetrize()`) that support the user for some specific choices.

scGraphVerse currently implements:

- **Assumption-free methods**: These methods use machine learning approaches to learn about gene relationships from the expression data without distributional assumptions.
  - **GENIE3**: Random forest-based method, already available in Bioconductor.
  - **GRNBoost2**: Gradient boosting implementation developed in the Arboreto framework (Python).
  - **JRF**: Joint Random Forest for multi-dataset inference, internally re-implemented following the Bioconductor guidelines.
- **Model-based methods**: These methods explicitly model the statistical properties of scRNA-seq data as zero-inflated negative binomial.
  - **ZILGM**: Zero-inflated local graphical models for sparse scRNA-seq data, internally re-implemented following the Bioconductor guidelines.
  - **PCzinb**: Partial correlation with zero-inflated negative binomial distribution, internally re-implemented following the Bioconductor guidelines.

The modular design of our implementation makes it easy to add new methods, enabling the inclusion of other algorithms in future releases.

The user can perform network inference using the main function `infer_networks()`, specifying the inference method. If the user provides a single (or merged) MAE object, scGraphverse will perform single-dataset (or early integration) using either GENIE3, GNRBoost2, ZILGM, or PCzinb methods. If the MAE object contains multiple datasets, scGraphverse will perform late integration using either GENIE3, GNRBoost2, ZILGM, or PCzinb, or joint integration using JRF, depending on the chosen method.

Note that the `infer_networks()` function returns different output formats depending on the inference method and the type of inference strategy; therefore, some additional steps might be required.

In particular,

- **Assumption-free methods** (GENIE3, GRNBoost2, JRF): Return a list of dataframes containing weighted gene-gene importance scores, where higher weights indicate stronger predicted regulatory relationships.
- **Model-based methods** (ZILGM, PCzinb): Return `SummarizedExperiment` object with adjacency matrices stored in the `assays` slots.

Therefore, for assumption-free methods, the user must convert the dataframes of importance scores into weighted, undirected adjacency matrices. This post-processing can be achieved in two simple steps:

1. **Generate adjacency matrix**: the `generate_adjacency()` function converts dataframes of weighted scores into a SummarizedExperiment containing the adjacency matrices.
2. **Symmetrize the network**: the `symmetrize()` function creates an undirected network representation by combining directional edges.

Instead, model-based methods return networks that are already symmetric and binary, so these conversion steps are not required.

1.4 Module 4: Edge Selection

The execution of this module is required only for assumption-free methods, to convert the undirected weighted adjacency matrices obtained in the Module 3 into binary matrices. Specifically, the `cutoff_adjacency()` function applies a permutation-based approach combined with quantile thresholding to identify statistically significant edges, thereby transforming weighted networks into binary adjacency matrices. To select the cutoff, the user must provide both the original MAE object (created in Module 1) and the weighted inferred networks (obtained from Module 3) as inputs to the `cutoff_adjacency()` function, specifying the same inference method and symmetrization parameter adopted in Module 3 and modulate the cutoff through the quantile_threshold parameter.

In contrast, since model-based methods produce networks that are already symmetric and binary, this step is not required.

1.5 Module 5: Consensus Network Building

At this stage, the output of scGRaphverse consists of K adjacency matrices. If $K > 1$, the user must aggregate the individual binary adjacency matrices into a unified network using the `create_consensus()` function after network inference and edge selection modules. Such a step is necessary for the late integration. Although JRF jointly estimates the adjacency matrices across datasets, it still produces K adjacency matrices. Hence, the user must create a consensus utilizing the same function after edge selection, also for JRF. Obviously, since the early integration

strategy or single-dataset inference directly provides a $K = 1$ adjacency matrix, the user does not need to execute this module, and the available adjacency matrix is treated as the consensus.

The current version of scGraphVerse provides three strategies:

- **Vote**: Edges present in the majority of networks (specified parameter)
- **Union**: Edges present in any network (high sensitivity)
- **INet**: Iterative network refinement

After building the consensus network, the user has all the necessary data to validate the inference strategy/methods and delve into network biology.

1.6 Module 6: Analysis & Validation

The scGraphVerse workflow ends with comprehensive tools for network validation and biological interpretation. The main output of the previous modules (center of Figure S1) is the consensus network, which represents the shared network across the datasets or the final inferred network for a single dataset. In addition to this main output, scGraphVerse also generates several intermediate network representations throughout the workflow. During late and joint integration, the workflow produces the individual networks corresponding to each dataset. All inference methods produce binary adjacency matrices stored in a SummarizedExperiment. For model-based methods, these binary matrices are returned directly at the end of Module 3, whereas for assumption-free methods, they are obtained after applying the cutoff procedure in Module 4. In addition to the binary networks, assumption-free methods also return weighted adjacency matrices during inference, which are provided as a SummarizedExperiment. Using these network objects, the user can explore both the performance of the inference process and the structural properties of the resulting networks in depth.

To support biological interpretation, scGraphVerse interfaces with STRINGdb to reconstruct a physical-biological interaction network, based on gene names. Using the `stringdb_adjacency()` function, users can query STRINGdb and generate an adjacency matrix containing the genes of interest used as input, specifying the threshold for the STRING confidence score and the organism. Whenever downstream analyses require a reference network, scGraphVerse can use either a user-provided network or generate one using the `stringdb_adjacency()` function.

1.6.1 Integration & Inference Performance Evaluation

To evaluate the performance of both the inference methods and the integration strategies, scGraphVerse implements three dedicated functions (Figure S1 purple box) that assess reconstruction accuracy with respect to a reference network:

- `plotROC()`: Generates Receiver Operating Characteristic (ROC) curves and computes the Area Under the Curve (AUC).
- `pscores()`: Computes edge-level metrics (TPR, FPR, Precision, F1, MCC, see Section 3 for the definitions of such indices).
- `compare_consensus()`: Compares inferred network against reference network.

plotROC() evaluates the weighted networks produced by assumption-free methods in Module 3, enabling the user to directly inspect the ROC curves and corresponding AUC values using the reference network as ground truth. Moreover, to evaluate the performance of integration and inference methods in reconstructing the reference, the user can apply the `pscores()` function, providing as input a SummarizedExperiment containing either multiple intermediate binary adjacency matrices or a single adjacency matrix of the final network (from consensus or early/single

integration). For visualization, the user can use the `compare_consensus()` function to plot the binary network, both visualizing and comparing it with the reference using a facet-plot or coloring it according to the presence or absence of edges in the reference network.

1.6.2 Network Visualization & Analysis

To explore and analyse the networks, scGraphVerse provides several functions (Figure S1 purple box) detailed in S2:

Table S2: Visualization and data exploration functions in scGraphVerse

Function	Input	Output	Dependencies
**Network Visualization**			
`plotg()`	SummarizedExperiment	ggplot object	`ggraph` Pedersen [2025], `igraph` Csardi and Nepusz [2006], `ggplot2` Wickham [2016]
**Performance Evaluation**			
`plotROC()`	SummarizedExperiment Ground truth matrix	ROC curves, AUC	`pROC` Robin et al. [2011], `ggplot2` Wickham [2016]
`pscores()`	SummarizedExperiment Ground truth matrix	Performance metrics	`fmsb` Nakazawa [2024], `dplyr` Wickham et al. [2023], `tidyr` Wickham et al. [2024]
**Network Validation**			
`edge_mining()`	SummarizedExperiment Ground truth matrix	Literature evidence	`BiocParallel` Morgan et al. [2025], `rentrez` Winter [2017]
`compare_consensus()`	SummarizedExperiment Ground truth matrix	Comparison plots	`ggplot2` Wickham [2016], `STRINGdb` Szklarczyk et al. [2023], `ggraph` Pedersen [2025]
**Biological Interpretation**			
`community_path()`	SummarizedExperiment	Communities pathways	`robin` Policastro et al. [2021, 2025], `clusterProfiler` Xu et al. [2024], Yu [2024], `DOSE` Yu et al. [2015]
`community_similarity()`	SummarizedExperiment Ground truth community	Similarity metrics	`igraph` Csardi and Nepusz [2006], `fmsb` Nakazawa [2024]

After assessing network inference performance, the user can visualize the network with the `plotg()` function. If the input SummarizedExperiment object contains multiple networks, the function automatically generates a facet-plot. To study networks from a biological perspective, scGraphVerse integrates community detection with pathway enrichment analysis, utilizing KEGG and REACTOME databases. Using the `community_path()` function, the user can perform both analyses simultaneously, obtaining a dataframe that reports the detected communities and their significantly enriched pathways.

Moreover, scGraphVerse visualizes the network by coloring nodes according to their community assignments. Using the output of the `community_path()` function, the user can further assess community structure and network topology by applying the `compute_community_metrics()` and `compute_topology_metrics()` functions, respectively. In addition, scGraphVerse provides

a wrapper function, `community_similarity()`, which computes community assignment metrics, evaluates topological properties, and optionally produces comparative visualizations.

Finally, the user can investigate the existing biological knowledge associated with the predicted edges. scGraphVerse performs automated literature mining by querying PubMed for keywords and abstracts related to each gene pair (edge). Using the `edge_mining()` function and providing a reference network, the user can annotate each edge in the input `SummarizedExperiment` with PubMed identifiers (PMIDs) and with information from the reference network, indicating whether an edge is present or absent in the reference and whether it is cited or not cited in the literature.

The user can combine multiple validation approaches to increase confidence in predicted networks. For a quick start and vignettes, the user can run the snippets and examples at https://ngsfc.github.io/scGraphVerse/.

1.7 Multimodal Integration and future extensions

scGraphVerse is designed to infer gene regulatory networks from scRNA-seq data. However, since it uses data structures compatible with the existing Bioconductor ecosystem, it can be easily integrated with other existing pipelines, including those designed for single-cell multimodal analysis, without requiring data conversion. Therefore, the availability of an additional multimodal layer or external data can be used with the current version of scGraphVerse in two ways.

- `Improving Cell type identification`. scGraphVerse performs network inference for each cell type of interest, assuming the cell types are already identified. Therefore, before using scGraphVerse, users can perform clustering using single-cell multimodal approaches, such as weighted nearest neighbor (WNN) analysis as implemented in Seurat (see, for example, the Seurat Multimodal Analysis vignette). Such approaches will identify a more accurate, biologically relevant cell population based on the available multimodal features (e.g., chromatin accessibility and/or surface protein abundance). After the clustering step, the RNA count assay for the selected cluster can be used as input to scGraphVerse's inference pipeline without any further format conversion, since scGraphVerse accepts Seurat, SingleCellExperiment, or plain-matrix objects.

- `Assessing Edges`. After the network inference step, the availability of multimodal data, such as scATAC-seq, can be used to qualitatively validate the inferred network edges. For example, using the Signac or the ArchR packages with scATAC-seq fragment files, it is possible to provide evidence for the chromatin accessibility of regulatory regions associated with inferred edges by visualizing read coverage of the selected target genes. In that case, multimodal scATAC-seq targeting the same cell type enables exploration of cluster-specific chromatin accessibility. To a lesser extent, bulk ATAC-seq data could also be used to better support the edges, in combination with the rtracklayer package. Although such additional data could be used to validate a specific interaction of interest, they do not play a role in inference.

Indeed, it should be noted that network inference could also be performed in a fully multimodal framework, modeling the different omics jointly as inputs to the inference algorithms. This approach would represent a distinct methodological problem that is not currently supported by scGraphVerse, but we want to consider it in the future.

2 Data Simulation

2.1 Ground Truth Network Construction

We constructed ground-truth networks based on experimentally validated biological interactions, ensuring that both the edges and the overall topology reflect the biological organization of ob-

served gene networks. We represented the ground truth network as a binary adjacency matrix $\mathbf{A}^* \in \{0,1\}^{p \times p}$, where p denotes the number of genes. Each element A^*_{ij} specifies the presence ($A^*_{ij} = 1$) or absence ($A^*_{ij} = 0$) of an edge between genes i and j. The matrix $\mathbf{A}^*$ satisfies the following mathematical properties:

$$A^*_{ij} = A^*_{ji} \quad \forall i, j \in \{1, \dots, p\} \quad \text{(symmetry)} \tag{1}$$

$$A^*_{ii} = 0 \quad \forall i \in \{1, \dots, p\} \quad \text{(no self-loops)} \tag{2}$$

$$\sum_{i<j} A_{ij} = |E| \quad \text{(edges count)} \tag{3}$$

where $|E|$ denotes the total number of edges in the network. To build $\mathbf{A}^*$, we extracted the protein-protein physical interactions from STRINGdb. Specifically, let $\mathcal{S} = \{(g_i, g_j, s_{ij})\}$ denote the set of all STRINGdb interactions, where g_i, g_j are gene identifiers and $s_{ij} \in [0, 1000]$ is the confidence score. We then defined

$$A^*_{ij} = \begin{cases} 1 & \text{if } (g_i, g_j, s_{ij}) \in \mathcal{S} \text{ and } s_{ij} \geq \tau_{conf} \\ 0 & \text{otherwise} \end{cases} \tag{4}$$

where $\tau_{conf} = 900$ represents our selected high-confidence threshold.

Figure S2 illustrates examples of the resulting networks generated for different values of p, corresponding to varying numbers of genes.

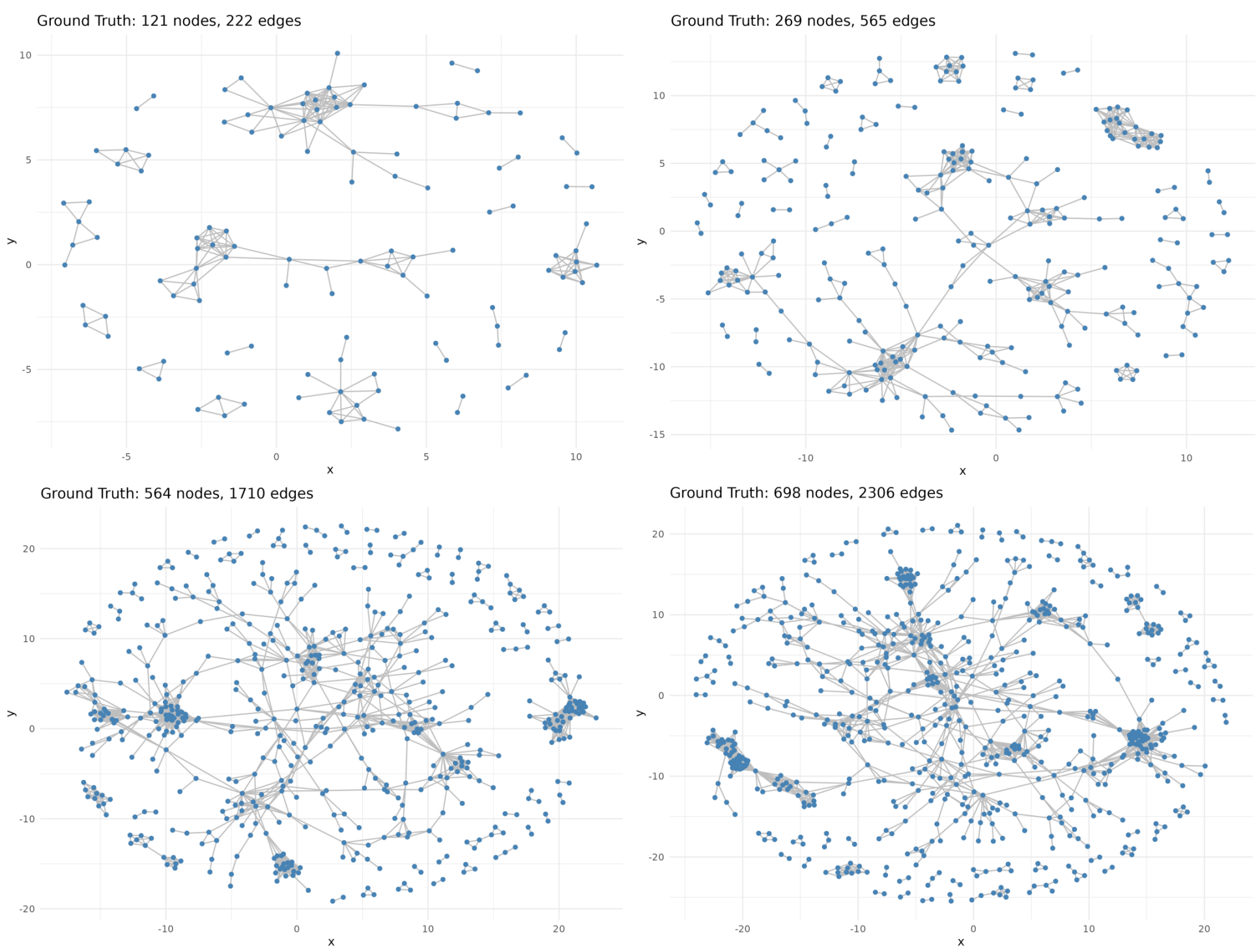

Figure S2: Ground-truth networks constructed from STRINGdb considering different numbers of genes.

2.2 Zero-Inflated Count Data Simulation

Given $\mathbf{A}^*$, we simulated count matrices through a graph-based construction aimed at reproducing salient features of scRNA-seq data, such as overdispersion, excess zeros, and sparsity patterns, rather than enforcing a strict multivariate ZINB model. First, we constructed an **incidence expansion matrix** $\mathbf{B} \in \{0,1\}^{p \times (p+|E|)}$ as:

$$\mathbf{B} = [\mathbf{I}_p \mid \mathbf{P}] \tag{5}$$

where $\mathbf{I}_p$ is the $p \times p$ identity matrix, $\mathbf{P}$ is a matrix with p rows and $|E|$ columns that encodes the interactions between genes. $\mathbf{P}$ contains zero entries and two elements equal to one in each column. For each interaction, the two elements equal to one are placed in the rows corresponding to the two genes that are connected in the network $\mathbf{A}^*$. Second, we generated a latent ZINB matrix $Y^{(k)}$ of dimension $n \times (p + |E|)$, with each latent variable $Y_{jl}^{(k)} \sim \text{ZINB}(\mu_l^{(k)}, \theta^{(k)}, \pi^{(k)})$. Here, the zero-inflation parameter $\pi^{(k)}$ models the dropout events in the k-th single-cell dataset, and $\mu_l^{(k)}$ and $\theta^{(k)}$ are the mean and the overdispersion parameter of the NB distribution, respectively. The parameter vector $\boldsymbol{\mu^{(k)}} = (\mu_l^{(k)})_{l=1,\ldots,(p+|E|)}$ represents the mean effect of individual genes and the interaction contributions in the k-th single-cell dataset. We then simulated the k-th dataset as $\mathbf{X}^{(k)} = \mathbf{Y}^{(k)}\mathbf{B}^T + \mathbf{N}^{(k)}$, where the matrix noise $\mathbf{N}^{(k)}$ of dimension $n \times p$ has entries $N_{jl}^{(k)} \sim \text{ZINB}(\mu_{noise}^{(k)}, 1, \pi^{(k)})$. Finally, we rescaled each dataset according to the sequencing depth, such that each cell j receives a depth factor $d_j \sim \text{Uniform}(d_{\min}, d_{\max})$ (Table S3).

Table S3: ZINB Simulation Parameters for the simulation study. p: number of genes, n: number of cells, K: number of datasets.

p - n scale ($p \in \{100, 200, 500, 700\}$, $n \in \{100, 500\}$, $K = 3$)

	$\boldsymbol{\mu}_l^{(k)}$	$\boldsymbol{\mu}_{noise}^{(k)}$	$\boldsymbol{\theta}^{(k)}$	$\boldsymbol{\pi}^{(k)}$
$\mathbf{X}^{(1)}$	U([1, 4])	1	1.0	0.2
$\mathbf{X}^{(2)}$	U([1, 7])	3	0.7	0.2
$\mathbf{X}^{(3)}$	U([1, 10])	5	0.5	0.2

($p = 500$, $n = 100$, $K = 5$)

	$\boldsymbol{\mu}_l^{(k)}$	$\boldsymbol{\mu}_{noise}^{(k)}$	$\boldsymbol{\theta}^{(k)}$	$\boldsymbol{\pi}^{(k)}$
$\mathbf{X}^{(1)}$	U([1, 4])	1	1.0	0.2
$\mathbf{X}^{(2)}$	U([1, 7])	3	0.7	0.2
$\mathbf{X}^{(3)}$	U([1, 10])	5	0.5	0.2
$\mathbf{X}^{(4)}$	U([1, 12])	6	0.3	0.2
$\mathbf{X}^{(5)}$	U([1, 15])	7	0.2	0.2

3 Performance Evaluation Framework

To evaluate the integration strategies when varying the number of genes (p), cells (n), and datasets (k) during the benchmarking, we implemented a multi-level performance assessment framework. We measure the performance at three distinct levels: edge-level, network-level, and community-level. Indices at the edge-level aim to assess the quality of the inferred network by examining the differences in edges with the ground-truth. Those at the network level aim to compare the statistical properties of the inferred network with those of the ground-truth. Finally, those at the community-level aim to validate the retained biological interpretation.

3.1 Edge-Level Performance Indices

With the Edge-Level measures, we quantify the accuracy of edge classification compared to the ground truth network, assessing how well the inferred network matches experimentally validated or literature-curated interactions. Let TP, TN, FP, and FN denote true positives (correctly predicted edges), true negatives (correctly predicted absent edges), false positives (incorrectly predicted edges), and false negatives (missed true edges), respectively. Considering these in-

dices, we provide the network reconstruction quality, emphasizing different aspects of prediction performance.

3.1.1 Sensitivity and Specificity

Sensitivity and specificity measure the ability to detect true interactions while avoiding false predictions:

$$\text{TPR (Sensitivity/Recall)} = \frac{TP}{TP + FN}. \tag{6}$$

$$\text{FPR (1 - Specificity)} = \frac{FP}{FP + TN}. \tag{7}$$

True Positive Rate (TPR), also known as sensitivity or recall, quantifies the proportion of edges that are successfully identified by the inference method. High sensitivity indicates a comprehensive identification of edges.

False Positive Rate (FPR) measures the proportion of truly absent edges that are incorrectly predicted as edges. In biological networks, where the number of potential edges ($p(p-1)/2$) usually exceeds actual connections, controlling FPR is critical. In fact, a low FPR can also generate several spurious edges, hiding the true biological signal.

3.1.2 F1-Score

F1-score provides a harmonic mean of precision and sensitivity, offering a balanced assessment between these complementary metrics. In general, the harmonic mean represents an excellent performance using only one index, since a higher F1-score indicates a good balance between the detection and the accuracy of the edges. However, in this context, it must be interpreted carefully since the positive and negative classes are not balanced by design.

3.1.3 Matthews Correlation Coefficient

The Matthews Correlation Coefficient (MCC) provides a balanced measure that accounts for all four confusion matrix components:

$$\text{MCC} = \frac{TP \cdot TN - FP \cdot FN}{\sqrt{(TP + FP)(TP + FN)(TN + FP)(TN + FN)}} \tag{8}$$

MCC ranges from -1 (perfect disagreement) to +1 (perfect agreement), with 0 indicating performance no better than random prediction. This metric is not affected by unbalanced datasets, where the true edges represent a small fraction of all possible edges. Consistent with this, MCC is more suitable than F1-score for comparing methods across different network sizes, sparsity levels, and inference strategies.

3.2 Network-Level Performance Measures

Network-level measures validate whether the inferred networks encode a meaningful organization with similar global statistical topological properties to those observed on real data. We compute three key topological indices to assess the global structure of inferred networks:

Density quantifies the proportion of actual edges relative to all possible edges in the network, defined as $D = \frac{2E}{N(N-1)}$ where E is the number of edges and N is the number of nodes. In biological networks, density reflects the overall connectivity and coordination between genes, which should not be excessive (resulting in too many false positives). In contrast, extremely sparse networks may miss important information.

Transitivity measures the tendency of nodes to form groups, calculated as the ratio of closed triplets (triangles) to all triplets in the network: $T = \frac{3 \times \text{number of triangles}}{\text{number of connected triplets}}$. Higher transitivity leads to a nice identification of regulatory modules..

Modularity assesses the degree to which a network can be divided into distinct communities or modules, with many edges within communities and few edges between them. Modularity is defined as $Q = \frac{1}{2E} \sum_{ij} \left[A_{ij} - \frac{k_i k_j}{2E} \right] \delta(c_i, c_j)$, where A_{ij} is the adjacency matrix, k_i and k_j are the degrees of nodes i and j, and $\delta(c_i, c_j) = 1$ if nodes i and j belong to the same community. A higher modularity indicates a strong community structure, reflecting the specialization of modules in distinct biological processes that involve a specific cell type.

3.3 Community Structure Performance Measures

Community structure measures evaluate the functional organization by comparing predicted and ground-truth communities. We employ three complementary information-theoretic indices:

1. **Normalized Mutual Information (NMI)**: Measures the shared information between predicted and true community structures while accounting for the number of communities. NMI is defined as:

$$\text{NMI}(C, \hat{C}) = \frac{2 \cdot I(C, \hat{C})}{H(C) + H(\hat{C})} \tag{9}$$

   where $I(C, \hat{C})$ is the mutual information between true communities C and predicted communities $\hat{C}$, and $H(\cdot)$ denotes entropy. NMI ranges from 0 (no shared information, completely different partitions) to 1 (perfect agreement).

2. **Adjusted Rand Index (ARI)**: Quantifies partition similarity using a chance-adjusted measurement, providing a robust comparison across different community detection algorithms. ARI is computed as:

$$\text{ARI} = \frac{\sum_{ij} \binom{n_{ij}}{2} - \left[\sum_i \binom{a_i}{2} \sum_j \binom{b_j}{2} \right] / \binom{n}{2}}{\frac{1}{2} \left[\sum_i \binom{a_i}{2} + \sum_j \binom{b_j}{2} \right] - \left[\sum_i \binom{a_i}{2} \sum_j \binom{b_j}{2} \right] / \binom{n}{2}} \tag{10}$$

   where n_{ij} is the number of nodes in both community i of the true partition and community j of the predicted partition, a_i and b_j are the sizes of communities in each partition, and n is the total number of nodes. ARI ranges from -1 to 1, with 1 indicating perfect agreement, 0 indicating random partitioning, and negative values indicating agreement worse than random.

3. **Variation of Information (VI)**: Measures information-theoretic dissimilarity between community compositions, offering a complementary perspective to similarity-based metrics. VI is defined as:

$$\text{VI}(C, \hat{C}) = H(C|\hat{C}) + H(\hat{C}|C) = H(C) + H(\hat{C}) - 2I(C, \hat{C}) \tag{11}$$

   where $H(C|\hat{C})$ represents the conditional entropy (information needed to describe C given $\hat{C}$). Unlike similarity metrics, VI measures distance between partitions and ranges from 0 (identical partitions) to $\log n$ (maximally different partitions).

Together, these three types of indices provide a comprehensive assessment of the benchmarking study.

4 Consensus approaches for late integration

scGraphVerse implements different consensus strategies for the late integration. In Figure S3, we report the performance of INet, Union, and Vote consensus strategies for the assumption-free node-wise methods (GENIE3, GRNBoost2) in both $n = 100$ (Figure S3, left panel) and $n = 500$ (Figure S3, right panel), varying from $p = 100$ to $p = 700$ (i.e., as in the second simulation scenario).

For $n = 100$, we observed that the "voting" approach achieves the highest Precision and a comparable MCC at $p = 100$. While at $n = 500$ through the p scaling, we reported an even better performance of the "voting" consensus method, achieving higher F1, MCC, and Precision. Considering this, in the benchmarking analysis (both $n = 100$ and $n = 500$), we considered the "voting" approach as the default consensus for the late integration strategy.

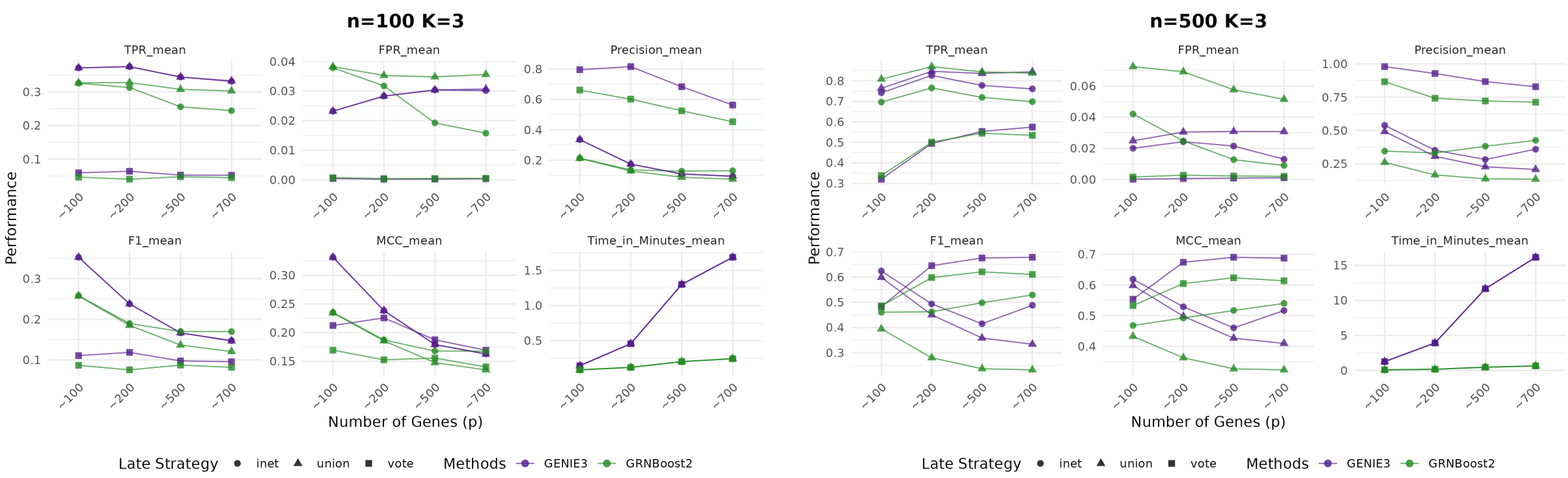

Figure S3: Comparison of late integration results with different sample sizes.

5 Case study 1: Edge mining

Performing edge mining, we found that all interactions involved genes present in community-enriched pathways, demonstrating functional coherence validated through PubMed literature mining (Table S4). Community 3 interactions support actin cytoskeleton dynamics, which are essential for B-cell migration and immune synapse formation. Community 7 highlights mitochondrial homeostasis. Community 9 emphasizes ER protein processing, which is crucial for the secretion of antibodies in plasma cells. ER: Endoplasmic Reticulum; UPR: Unfolded Protein Response.

6 Case study 2: Medulloblastoma network inference

We downloaded the scRNA-seq count matrices from two medulloblastoma patients (SCPCS000136 and SCPCS000139). After selecting the top 3.000 expressed genes using `selgene()` and excluding mitochondrial genes, ribosomal genes, and MALAT1, we inferred the networks with GENIE3 (15 cores) under two strategies: late integration and early integration. Weighted adjacency matrices were symmetrised and binarised at the 99th percentile edge-weight quantile with $n = 5$. Using `community_path()`, we performed community detection using the Louvain modularity-maximisation algorithm. Selecting for the Communities containing at least 5 genes, we explore the KEGG pathways using clusterProfiler, keeping as biological interpretation the terms with adjusted $p < 0.05$ S4.

Table S4: Top Literature-Supported Interactions by Community

Community	Enriched Pathways	Interaction	Citations	Biological Function
Community 3	Regulation of actin cytoskeleton, Tight junction	CFL1-PFN1	15	Actin dynamics regulation
		MYL12A-MYL12B	6	Cell contractility and motility
		ARPC2-PFN1	4	Arp2/3 complex-mediated branching
		ARPC2-CFL1	2	Actin filament nucleation
		PFN1-TMS4X	2	Membrane-cytoskeleton linkage
Community 7	Mitophagy - animal	TOMM7-UBA52	1	Mitochondrial protein
Community 9	Protein processing in endoplasmic reticulum	CALR-HSPA5	22	ER stress and unfolded protein response
		DAD1-HSPA5	13	ER-associated protein quality control
		HSPA5-SEC61B	8	Protein translocation into ER lumen
		ERP29-HSPA5	2	ER chaperone network coordination
		SPCS1-SPCS2	2	Signal peptidase complex activity

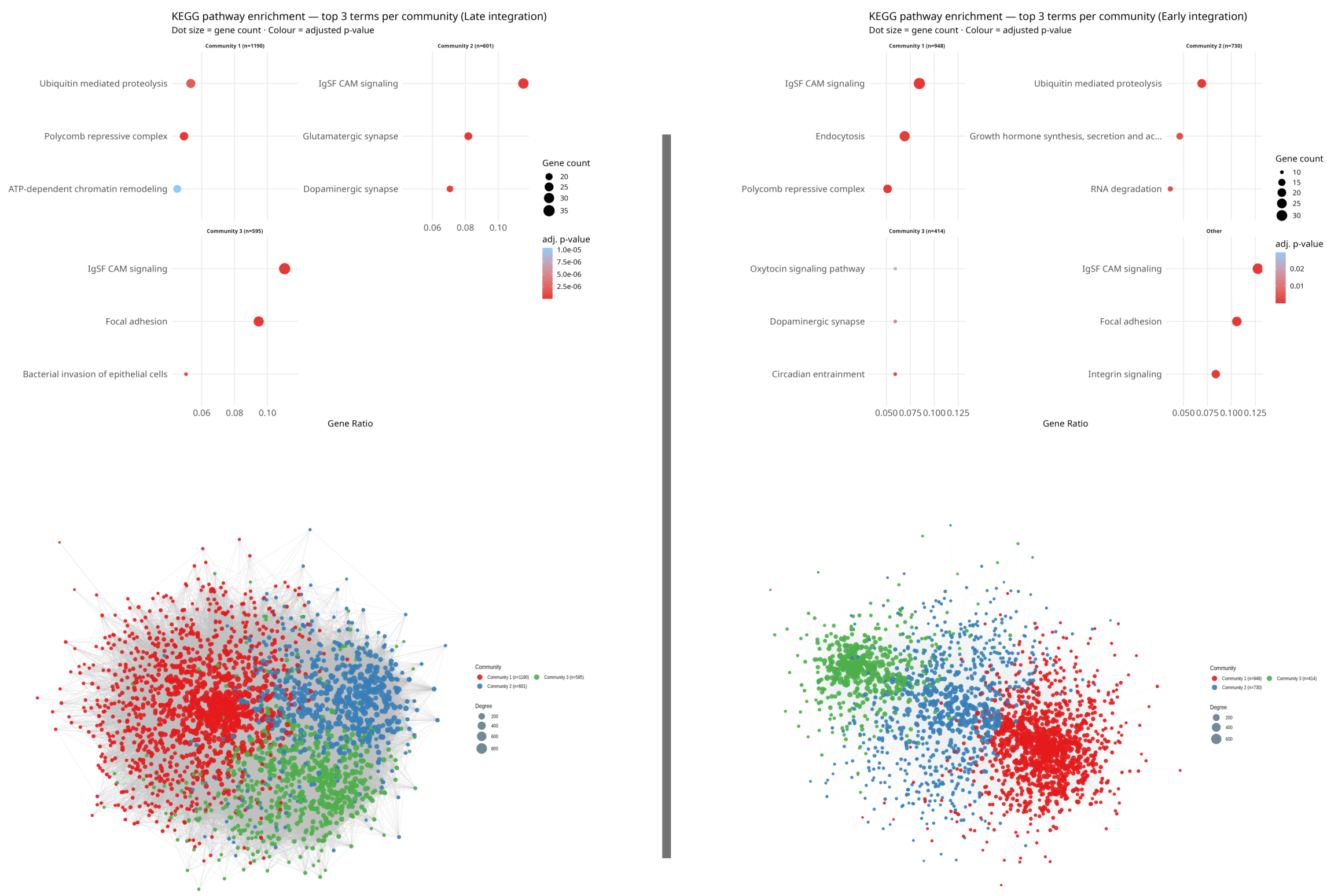

Figure S4: Medulloblastoma datasets network inference results. Upper the enriched pathways for communities using both late and early integration strategies (left and right rispectively). Down network representation of the communities identified.

References

G. Csardi and T. Nepusz. The igraph software package for complex network research. *InterJournal*, Complex Systems:1695, 2006.

M. Morgan, J. Wang, V. Obenchain, M. Lang, R. Thompson, and N. Turaga. *BiocParallel: Bioconductor facilities for parallel evaluation*, 2025. URL https://bioconductor.org/packages/BiocParallel. R package version 1.42.1.

M. Nakazawa. *fmsb: Functions for Medical Statistics Book with some Demographic Data*, 2024. URL https://CRAN.R-project.org/package=fmsb. R package version 0.7.6.

T. L. Pedersen. *ggraph: An Implementation of Grammar of Graphics for Graphs and Networks*, 2025. URL https://ggraph.data-imaginist.com. R package version 2.2.2.9000.

V. Policastro, D. Righelli, A. Carissimo, L. Cutillo, and I. De Feis. Robustness in network (robin): an r package for comparison and validation of communities. *The R Journal*, 13:292–309, 2021.

V. Policastro, D. Righelli, L. Cutillo, and A. Carissimo. robin2: accelerating single-cell data clustering evaluation. *Bioinformatics Advances*, 2025. in press.

X. Robin, N. Turck, A. Hainard, N. Tiberti, F. Lisacek, J. C. Sanchez, and M. Müller. proc: an open-source package for r and s+ to analyze and compare roc curves. *BMC Bioinformatics*, 12:77, 2011.

D. Szklarczyk, R. Kirsch, M. Koutrouli, K. Nastou, F. Mehryary, R. Hachilif, A. L. Gable, T. Fang, N. T. Doncheva, S. Pyysalo, P. Bork, L. J. Jensen, and C. von Mering. The string database in 2023: protein-protein association networks and functional enrichment analyses for any sequenced genome of interest. *Nucleic Acids Research*, 51:D638–46, 2023.

H. Wickham. *ggplot2: Elegant Graphics for Data Analysis*. Springer-Verlag, New York, 2016.

H. Wickham, R. François, L. Henry, K. Müller, and D. Vaughan. *dplyr: A Grammar of Data Manipulation*, 2023. URL https://CRAN.R-project.org/package=dplyr. R package version 1.1.4.

H. Wickham, D. Vaughan, and M. Girlich. *tidyr: Tidy Messy Data*, 2024. URL https://CRAN.R-project.org/package=tidyr. R package version 1.3.1.

D. J. Winter. rentrez: An r package for the ncbi eutils api. *The R Journal*, 9:520–6, 2017.

S. Xu, E. Hu, Y. Cai, Z. Xie, X. Luo, L. Zhan, W. Tang, Q. Wang, B. Liu, R. Wang, W. Xie, T. Wu, L. Xie, and G. Yu. Using clusterprofiler to characterize multiomics data. *Nature Protocols*, 19:3292–320, 2024.

G. Yu. Thirteen years of clusterprofiler. *The Innovation*, 5:100722, 2024.

G. Yu, L. G. Wang, G. R. Yan, and Q. Y. He. Dose: an r/bioconductor package for disease ontology semantic and enrichment analysis. *Bioinformatics*, 31:608–9, 2015.
